# Corrosion mitigation for steel in acid environment using novel *p*-phenylenediamine and benzidine coumarin derivatives: synthesis, electrochemical, computational and SRB biological resistivity

**DOI:** 10.1039/d2ra05803k

**Published:** 2022-10-13

**Authors:** Hani M. Elaryian, Mahmoud A. Bedair, Ahmed H. Bedair, Rabab M. Aboushahba, Abd El-Aziz S. Fouda

**Affiliations:** Department of Chemistry, Faculty of Science (Men's Campus), Al-Azhar University Nasr City 11884 Cairo Egypt mbedair@ub.edu.sa mbedier@azhar.edu.eg m_bedier@yahoo.com; Zohr Gas Field, Belayim Petroleum Company Nasr City 7074 Cairo Egypt; College of Science and Arts, University of Bisha P.O. Box 101 Al-Namas 61977 Saudi Arabia; Department of Chemistry, Faculty of Science (Girls' Branch), Al-Azhar University, Nasr City 11574 Cairo Egypt; Department of Chemistry, Faculty of Science, Mansoura University Mansoura-35516 Egypt asfouda@hotmail.com asfouda@mans.edu.eg

## Abstract

Three novel *p*-phenylenediamine and benzidine coumarin derivatives were synthetized, namely: 4,4′-((((1,4-phenylenebis(azaneylylidene))bis(ethan-1-yl-1-ylidene))bis(2-oxo-2*H*-chromene-3,6-diyl))bis(diazene-2,1-diyl))dibenzenesulfonic acid (PhODB), 4,4′-(((-([1,1′-biphenyl]-4,4′-diylbis(azaneylylidene))bis(ethan-1-yl-1-ylidene))bis(2-oxo-2*H*-chromene-3,6-diyl))bis(diazene-2,1-diyl))dibenzenesulfonic acid (BODB) and 4,4′-(((-((3,3′-dimethoxy-[1,1′-biphenyl]-4,4′-diyl)bis(azaneylylidene))bis(ethan-1-yl-1-ylidene))bis(2-oxo-2*H*-chromene-3,6-iyl))bis(diazene-2,1-diyl))dibenzenesulfonic acid (DODB). Their chemical structures were proved by performing Fourier-transform infrared spectroscopy, proton nuclear magnetic resonance and mass spectrometry analysis. The synthesized *p*-phenylenediamine and benzidine coumarin derivatives were tested as corrosion inhibitors for mild steel (MS) in 1 M HCl solution using weight loss, electrochemical, morphological, and theoretical studies. The compound 3,3′-dimethoxy benzidine coumarin derivative (DODB) was proved to give the highest efficiency with 94.98% obtained from weight loss measurements. These compounds are mixed inhibitors, as seen by the polarization curves. Impedance diagrams showed that when the concentration of these derivatives rose, the double-layer capacitance fell and the charge transfer resistance increased. Calculated thermodynamic parameters were computed and the mechanism of adsorption was also studied for the synthesized *p*-phenylenediamine and benzidine coumarin derivatives. The ability of the synthesized derivatives to protect the surface against corrosion was investigated by scanning electron microscope (SEM), UV-visible spectroscopy and energy dispersive X-ray spectroscopy (EDX). Theoretical chemical calculations (DFT) and biological resistivity (SRB) were investigated.

## Abstract 2

Three novel *p*-phenylenediamine and benzidine coumarin derivatives were synthetized (PhODB), (BODB) and (DODB), these compounds showed excellent anti corrosion effect for steel in 1.0 M HCl and significant SRB resistance.

## Graphical abstract

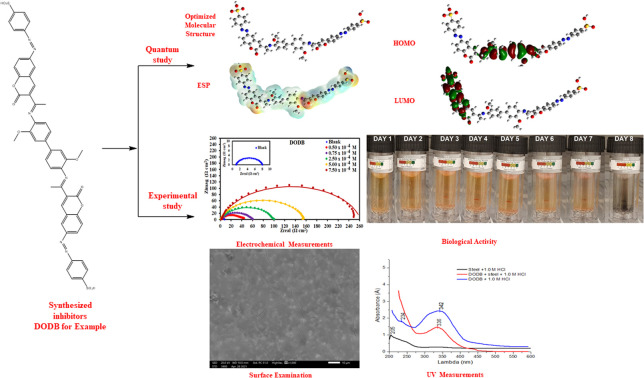

## 1. Introduction

The corrosion process is considered an electrochemical process performed naturally by the tendency of the metal to become more stable by conversion to its corresponding oxide. The use of metals is very widespread in the petroleum industry, pipelines, petrochemicals, power stations, turbines, and boilers. Using acids to remove the scales and rust formed on the metal surface during different operations is a very destructive process. The corrosion process can be mitigated by using inhibitors.^1^ Organic compounds with heteroatoms are preferred as corrosion inhibitors because they have good ability for electron donation.^2^ The ability of organic inhibitors to be more effective in the inhibition process increases when they contain the heteroatoms sulfur (S) and nitrogen (N), and sulfur (S) gives them the highest ability for electron donation.^3^ Electron donation to the d orbital of a metal is easier for organic compounds with available electrons to share located on their double bonds or *via* hereto atoms and this donation makes organic compounds potential corrosion inhibitors.^4–8^ Diethyl(phenyl(phenylamino)methyl)phosphonate (DEPAMP) and diethyl((2-methoxyphenyl)(phenylamino)methyl)phosphonate (*o*-DEPAMP), when utilized as corrosion inhibitors for XC48 steel in a 1 M HCl solution, yield 89.27% and 90.72% inhibition efficiencies at 10^−3^ M, respectively.^9^ 2,6-Bis(hydroxymethyl)-4-methoxyphenol (1) or 4-chloro-2,6-bis(hydroxymethyl)phenol (2) showed 93% and 84% inhibition efficiency at 5 × 10^−2^ M, respectively.^10^ When utilized as a corrosion inhibitor for XC48 carbon steel in 0.5 M H_2_SO_4_ solutions, (*E*)-1-(3-nitrobenzylidene)-2-(*p*-tolyl) hydrazine (*E*-NBPTH) demonstrated an inhibitory efficiency of 86.52% at 10^−3^ M.^11^ When used as corrosion inhibitors for XC48 carbon steel in 0.5 M H_2_SO_4_ solutions, (*E*)-*N*,*N*-dimethyl-4-((phenylimino)methyl)aniline (*E*-NDPIMA) and diethyl((4-(dimethylamino)phenyl)(phenylamino)methyl)phosphonate (α-APD) yielded respective results of 85.83% and 92.81% at 10^−3^ M.^12^ When used as a corrosion inhibitor for carbon steel in 0.5 M H_2_SO_4_ solution, 4-(2-[ethoxy(hydroxy)phosphonyl](3-nitrophenyl)methylhydrazinyl) benzoic acid achieved 88.63% inhibition at 10^−3^ M.^13^ Derivatives of quinoxaline, indole, benzimidazole and asphenyl-benzothiazole are various types of inhibitors used previously as potential corrosion inhibitors.^8,14–17^ Organic inhibitors are considered the best choice due to their easy synthesis, low cost, low toxicity, high purity and environmental friendliness among other advantages.^18,19^ Coumarins and their derivatives are organic compounds containing heterocycles, heteroatoms, double bonds and aryl rings with high ability for electron donation and use as potential corrosion inhibitors. Compounds containing an azo double bond can donate electrons to the metal surface and form a complex with it.^20,21^ The use of coumarin and its derivatives is widespread now because of their stability, availability and ability to donate electrons.^22,23^ Coumarin derivatives are widely used antibacterial, antifungal and antimicrobial, anti-inflammatory, anti-coagulant and antitumor agents. Furthermore, due to the green property of coumarin derivatives, they are also used as fixative and flavouring agents.^24–29^ In oil and gas industries, a common reason for pitting corrosion is microbial-influenced corrosion (MIC).^30^ The capacity for MIC increases as the deposits and accumulations in tubes and pipelines increase due to cathodic depolarization and galvanic cell formation.^31^ Twenty percent of corrosion costs are caused by microbial corrosion.^32,33^ Sulfate-reducing bacteria are the main microorganism responsible for sulfide generation.^34,35^*Desulfotomaculum* and *Desulfovibrio* strains are the most familiar SRB strains. These strains have a great ability to survive even in aggressive conditions like high pressure (507 bar), high temperature (40 °C) and also different pH values (4–8).^36^ Hydrogen sulfide gas (H_2_S), sulfate and metal sulfides are the most commonly generated products for SRB *via* an oxidation–reduction mechanism. Hydrogen sulfide gas (H_2_S) is liberated with sufficient concentration through this oxidation–reduction mechanism and drives the electrochemical process, leading to a localized fatigue corrosion mechanism.^37,38^ Eco-friendly organic compounds with biocidal properties are used in the petroleum industry and are added to decrease the bio corrosion process.^39^ The presence of biofilm causes resistance to the transfer of heat in heat exchangers and cooling towers.^40^

In the current study, novel *p*-phenylenediamine and benzidine coumarin derivatives were synthesized. The corrosion mitigation aptitude for the new organic coumarin derivatives to prevent steel corrosion in 1 M hydrochloric acid was examined by electrochemical methods and weight loss. Furthermore, morphological examination, DFT theoretical computational studies, UV-visible studies and action against SRB bacteria were also carried out.

## 2. Experimental techniques

### 2.1. Electrolytes and electrodes

Concentrated hydrochloric acid 37% (Merck) was diluted using demineralized pure water to prepare the required solution from (1.0 M) hydrochloric acid. Then, the diluted hydrochloric acid (1.0 M) was used for the preparation of multiple molar concentrations according to the molecular weight of each coumarin derivative. Due to the spontaneous corrosion process for the selected corrosive electrolyte, no stimulus or shaking was needed to proceed.

The dimensions of the mild steel specimen used in the weight loss measurements were 2.5 × 0.3 × 6 cm and the total area was 35.1 cm^2^. The wt% composition was Fe = 99.10, Mn = 0.45, Si = 0.25, C = 0.11, S = 0.05 and P = 0.04. In order to remove the undesired layer on the steel specimen surface, various emery paper grades (80–2000) were used for cleaning and polishing. Then, demineralized pure water and acetone were used for washing, and the specimens were dehydrated using a desiccator before performing the experimental procedures.

### 2.2. Synthesis of *p*-phenylenediamine and benzidine coumarin derivative inhibitors

**Fig. 1:**
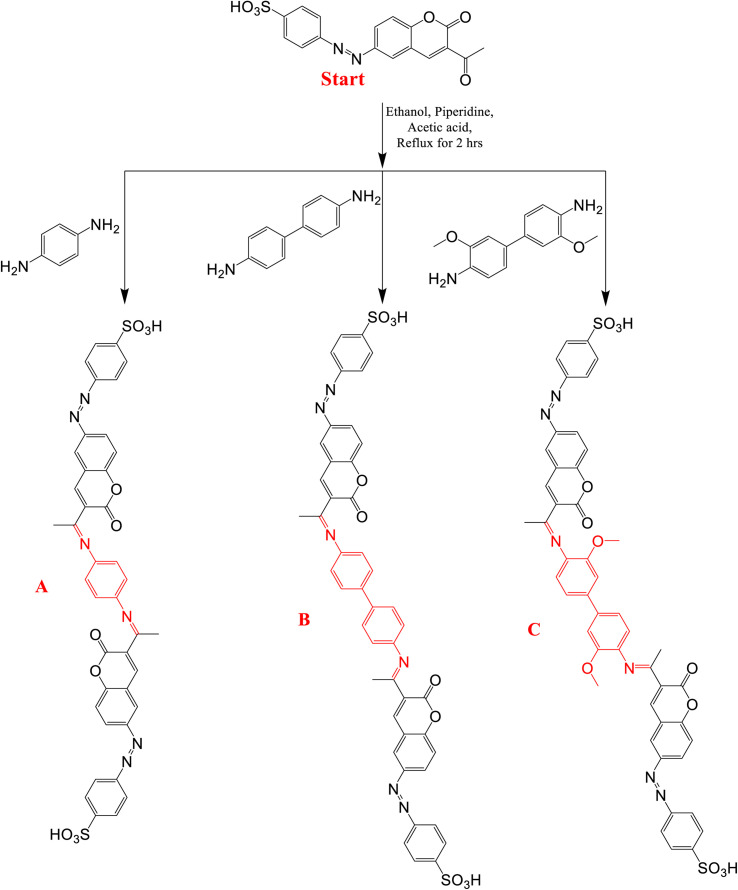
Preparation scheme for synthesized coumarin derivatives: (A) PhODB, (B) BODB and (C) DODB.

Using the previously synthesized acetyl nucleus from our previous study,^41^ three *p*-phenylenediamine and benzidine coumarin derivatives were prepared, as shown in Fig. 1. One mole each of *p*-phenylenediamine, benzidine and 3,3′-dimethoxy benzidine were reacted with two moles of 4-((3-acetyl-2-oxo-2*H*-chromen-6-yl)diazenyl)benzenesulfonic acid (Start), respectively. All reactions were performed in ethanol as solvent with a few drops of piperidine and glacial acetic acid as catalysts under refluxing for 2 h. The resulting products are 4,4′-((((1,4-phenylenebis(azaneylylidene))bis(ethan-1-yl-1-ylidene))bis(2-oxo-2*H*-chromene-3,6-diyl)) bis(diazene-2,1-diyl))dibenzenesulfonic acid (PhODB), 4,4′-(((-([1,1′-biphenyl]-4,4′-diylbis(azaneylylidene))bis(ethan-1-yl-1-ylidene))bis(2-oxo-2*H*-chromene-3,6-diyl))bis(diazene-2,1-diyl))dibenzenesulfonic acid (BODB) and 4,4′-(((-((3,3′-dimethoxy-[1,1′-biphenyl]-4,4′-diyl)bis(azaneylylidene))bis(ethan-1-yl-1-ylidene))bis(2-oxo-2*H*-chromene-3,6-iyl))bis(diazene-2,1-diyl))dibenzenesulfonic acid (DODB), respectively. Washing, drying and recrystallization were performed for all products. The products were solid powders with an orange reddish color, orange to brown color and rose to brown color for (PhODB), (BODB) and (DODB), respectively. The melting point was >300 °C for all the synthesized derivatives and sufficient yields were achieved, with 82.71%, 82.57% and 65.16% for (PhODB), (BODB) and (DODB), respectively.

### 2.3. Electrochemical measurements

In an electrochemical cell, a mild steel electrode with a (1 cm^2^ area) flat surface restrained using an epoxy holder was used as the working electrode (WE). A saturated calomel electrode (SCE) was used as the reference electrode and the counter electrode (CE) was made from graphite. The working electrode was exposed to 100 ml of acid electrolyte with different concentrations. The electrochemical analysis was carried out using a potentiostat/galvanostat/ZRA (Gamry-3000) analyzer with Gamry framework software for data acquisition (version 7.8.2). Fitting, plotting and graphing of the output data were determined with Gamry Echem Analyst software (version 7.8.2). Before performing every electrochemical test, the WE was first immersed in acid electrolyte for 3600 s to reach a stable steady state for the open circuit potential (OCP). The adjusted values for measuring the electrochemical impedance spectroscopy (EIS) were very low voltage (10 mV) with frequency range 100 kHz to 0.01 mHz and 10 points per decade at 25 °C. The adjusted potential values for potentiodynamic polarization measurements (PDP) ranged from −500 mV to 500 mV with a 1 mV s^−1^ scan rate at 25 °C. The adjusted frequency values for electrochemical frequency modulation (EFM) were 2 Hz and 5 Hz at 25 °C. The inhibition efficiency was calculated for EFM, EIS and PDP from the following equations:^42–44^1
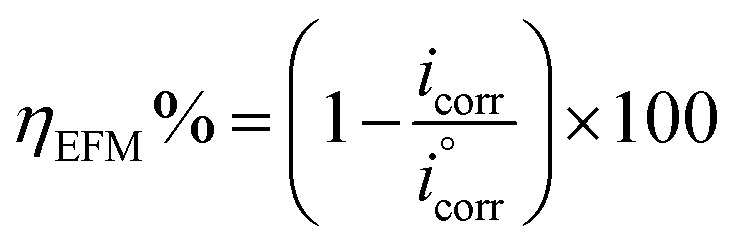
2
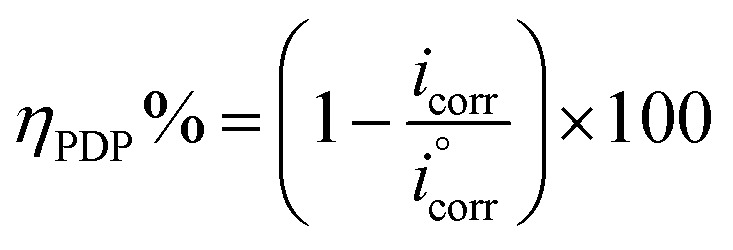
where, 
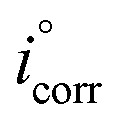
 and *i*_corr_ are current density for corrosion electrolytes without and with inhibitors, respectively.3
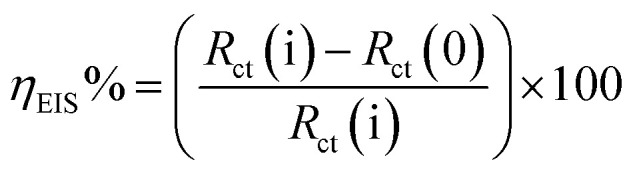
where, *R*_ct_(i) and *R*_ct_(0) are the charge transfer resistance with and without inhibitor, respectively, using 1.0 M HCl electrolyte medium.

### 2.4. Gravimetric weight loss measurements

After carefully cleaning the mild steel (MS) coupons, weight loss (WL) measurements were performed in 100 ml of corrosive electrolyte with and without inhibitors. WL was investigated at various temperatures (298, 303, 308, 313 and 318 K) using a water bath. Emery papers of various grades (80–2000) were used for cleaning and abrading the MS coupons. Demineralized pure water followed by acetone were used for the cleaning and washing steps and then the MS coupons were dried before starting the measurements. The exposure period for an MS coupon was 24 h of immersion in 100 ml of 1.0 M HCl corrosive electrolyte. The process of exposing the MS to 1.0 M HCl corrosive electrolyte was repeated using different concentrations of inhibitors dissolved in the same corrosive electrolyte (1.0 M HCl).

The corrosion rate was obtained using the following formula:

4 CR = Δ*W*/*At*

where, Δ*W* = (*W*_1_ (at initial time) − *W*_2_ (after 24 h)) in mg, *A* is the (MS) coupon surface area in cm^2^, *t* is the exposure time (h) and the overall units for the resulting value are mg cm^−2^ h^−1^.^45^

The calculation of (WL) *η*_WL_% inhibition efficiency can be performed using the following equations:

5 *θ* = (*W*_0_ − *W*_i_)/*W*_0_

6 *η*_WL_% = (*W*_0_ − *W*_i_/*W*_0_) × 100

where, *θ* = surface coverage, *W*_0_ = WL value without an inhibitor and *W*_i_ = WL value with an inhibitor.

### 2.5. Spectral surface analysis: UV-visible, SEM and EDX

After 24 h of exposure to a corrosive electrolyte at room temperature, a UV-vis spectrophotometer (Thermo Fisher Scientific) was used to prove complex formation between the synthesized coumarin derivatives and MS cations by measuring the changes in the wavelength values. Surface morphological examination was carried out with SEM-EDX (JEOL JSM-IT200 SEM). MS coupons with dimensions of 2.5 cm × 2.5 cm × 0.3 cm were abraded, scratched and cleaned using emery papers of various grades (1000–2000) before exposure to the corrosive electrolyte for 24 h before examination. After 24 h and before the examination, the MS coupons were washed with demineralized water and dehydrated. The MS coupon was fixed in the sample holder and SEM analysis was performed with (1000×) magnification to obtain a good detailed image of the examined (MS) coupon. EDX was used to measure the organic elements deposited from the synthesized derivatives on the MS surface which can prove surface protection *via* complexation between the synthesized derivatives and MS cations.

### 2.6. Bio-corrosion mitigation

Hydrogen sulfide (H_2_S) can be easily generated by sulfate-reducing (SRB) bacteria. Liberation of H_2_S increases the fatigue damage caused by corrosion. SRB (SRB-BART™ – DBI) vials were selected to monitor the bacterial growth of SRB, due to the low test period (11 days max) and high approximate population results. Once the vial has turned black, the test is complete and the SRB population can be recognized. The results can be achieved within eleven days, which is the maximum period for the test time. Each single day represents a specified quantity of SRB present and the test is complete when the first black sign appears on the test vial.^41^

### 2.7. Quantum chemical computation

Optimization of PhODB, BODB and DODB molecules was performed with semi-empirical (PM6), Hartree–Fock (631G) and DFT (6311G) basis set methods. DFT was used with 3 exchange function parameters for Beck's (B3LYP – Lee–Yang–Parr) correlation. Recently, DFT has been preferred due to its accuracy.^46^ The calculations were performed using the Gaussian 09 and Gauss View 06 packages.^47^*E*_HOMO_ and *E*_LUMO_ are known as the energies of the frontier molecular orbital (FMO), where *E*_HOMO_ and *E*_LUMO_ refer to the highest occupied and the lowest unoccupied molecular orbitals, respectively. According to Koopman's theory, the energy values for *E*_HOMO_ and *E*_LUMO_ can be expressed with other values like the energy gap (Δ*E*), ionization potential (IP), electron affinity (EA), electronegativity (*χ*), electrophilicity (*ω*), transferred electrons (ΔN), softness (*σ*), hardness (*η*), dipole moment (μ), total energy *E*(RB3LYP), molecular volume (MV) and total negative charge (TNC).^48^ The values of the abovementioned parameters can be calculated from the following equations:^49–54^

7 Δ*E* = *E*_LUMO_ − *E*_HOMO_

8 EA = −*E*_LUMO_

9 IP = −*E*_HOMO_

10
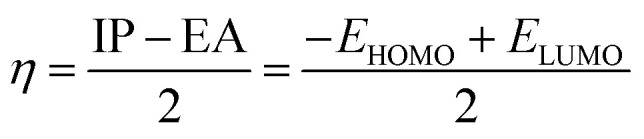

11 *χ* = (IP + EA)/2

12
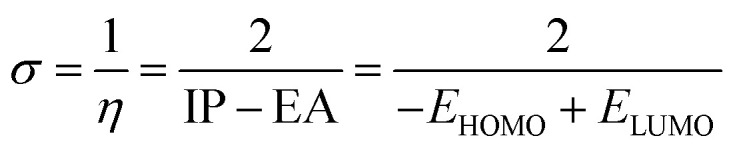
13
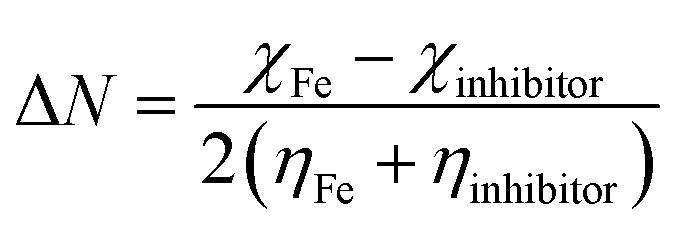
where the theoretical values are *χ*_Fe_ = 7.0 eV and *η*_Fe_ = zero,

14 *χ* = 0.5(LUMO + HOMO)

15 *ω* = (*χ* × *χ*)/2*η*

## 3. Results and discussion

### 3.1. Confirmation of the synthesis of *p*-phenylenediamine and benzidine coumarin derivatives

**Fig. 2:**
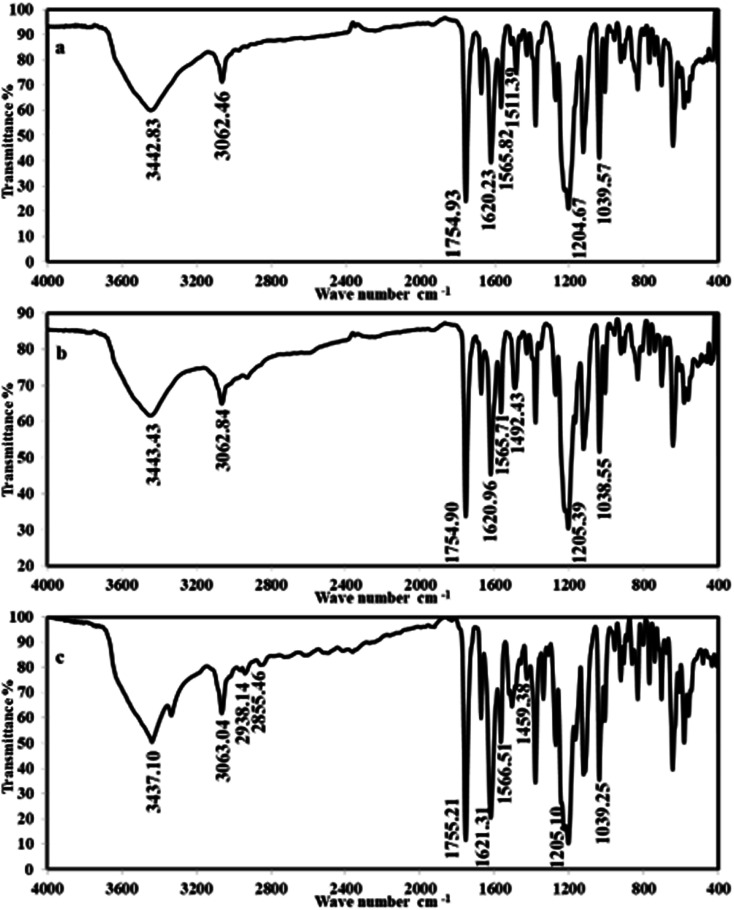
FTIR spectra for synthesized coumarin derivatives: (a) PhODB, (b) BODB and (c) DODB.

**Fig. 3:**
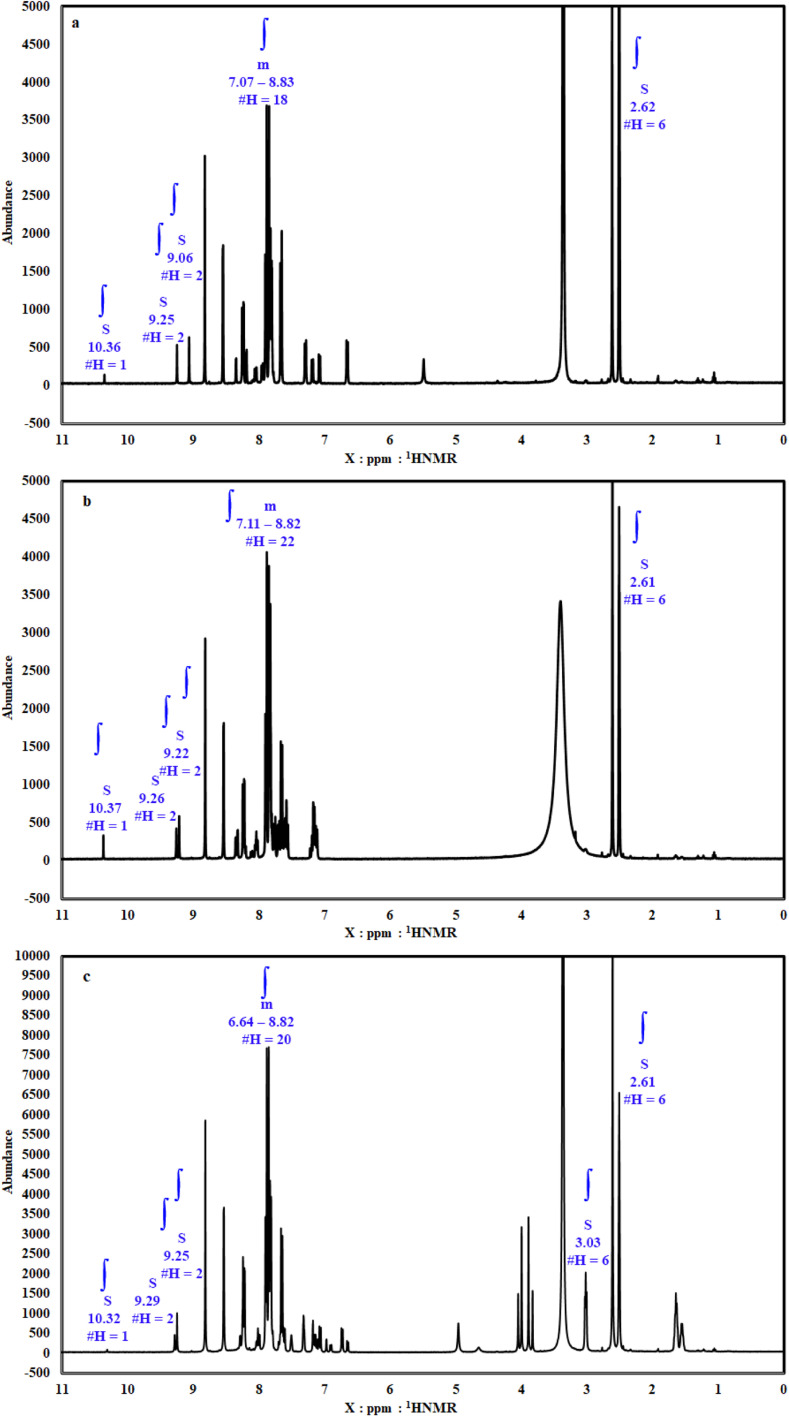
^1^H NMR spectra for synthesized coumarin derivatives: (a) PhODB, (b) BODB and (c) DODB.

**Fig. 4:**
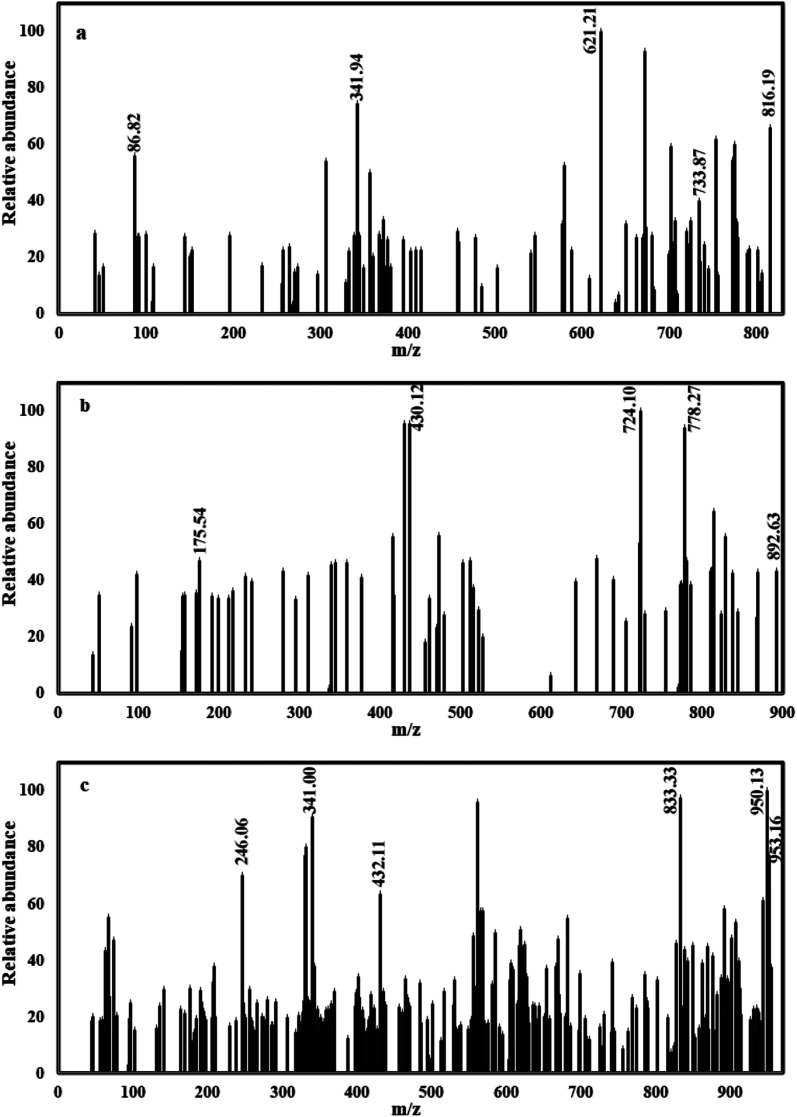
Mass spectra for synthesized coumarin derivatives: (a) PhODB, (b) BODB and (c) DODB.

The chemical structures for *p*-phenylenediamine and benzidine coumarin (PhODB, BODB and DODB) derivatives were confirmed by performing Fourier-transform infrared spectroscopy, proton nuclear magnetic resonance and mass spectrometry analysis. The FTIR of PhODB showed peaks at 3062.46 (**aromatic C–H**), 1754.93 (**C=O**), 1511.39, 1565.82 (**N=N**, azo groups, bis azo compound), 1204.67, 1039.57 (*δ* lactone, **O–C=O**), 1620.23 (**C=N**), and 3442.83 (**SO**_**3**_**H** group). The FTIR of BODB showed peaks at 3062.84 (**aromatic C–H**), 1754.90 (**C=O**, coumarin), 1492.43, 1565.71 (**N=N**, azo groups, bis azo compound), 1620.96 (**C=N**), 1205.39, 1038.55 (*δ* lactone, **O–C=O**), and 3443.43 (**SO**_**3**_**H** group). The DODB compound showed FTIR peaks at 3063.04 (**aromatic C–H**), 2855.46, 2938.14, 2962.67 (**aliphatic C–H**), 1755.21 (**C=O**), 1459.38, 1566.51 (**N=N**, azo groups, bis azo compound), 1205.10, 1039.25 (*δ* lactone, **O–C=O**), 1621.31 (**C=N**), 3437.10 (**SO**_**3**_**H** group). All detailed FTIR values are given in Table 1 and Fig. 2. From the ^**1**^**H NMR** analysis (**DMSO-*d***_***6***_), 400 MHz; PhODB showed bands at *δ* = 2.62 (6H, s, N=C–C**H**_**3**_), *δ* = 10.36 ppm (**SO**_**3**_**H**), *δ* = 7.07–8.83 ppm (18H, m, **Ar-H**), *δ* = 9.06, 9.25 ppm (2H, s, **coumarin-4-H**). But BODB showed bands at *δ* = 2.61 (6H, s, N=C–C**H**_**3**_), *δ* = 10.37 ppm (**SO**_**3**_**H**), *δ* = 7.11–8.82 ppm (22H, m, **Ar-H**), *δ* = 9.22, 9.26 ppm (2H, s, **coumarin-4-H**). The ^**1**^**H NMR** of DODB showed bands at *δ* = 2.61 (6H, s, N=C–C**H**_**3**_), *δ* = 3.03 (6H, s, **CH**_**3**_**–O-Ar**), *δ* = 10.32 ppm (**SO**_**3**_**H**), *δ* = 6.64–8.82 ppm (20H, m, **Ar-H**), *δ* = 9.25, 9.29 ppm (2H, s, **coumarin-4-H**). All detailed ^1^H NMR values are given in Table 1 and Fig. 3. From **mass** analysis at *m*/*z* (%); (**M**^**+.**^) = 816 (66.03%) for PhODB, (**M**^**+**^**˙**) = 892 (43.42%) for BODB and (**M**^**+**^**˙**) = 952 (66.15%) for DODB. The molecular ion peaks (base peak) are at *m*/*z* = 621 (100%) for PhODB, *m*/*z* = 724 (100%) for (BODB) and *m*/*z* = 950 (100%) for DODB. The other additional peaks are listed in Table 1 and Fig. 4.

**Table 1:** FTIR, ^1^H NMR and mass spectroscopy values for synthesized coumarin derivatives (PhODB, BODB and DODB)

Assignment	PhODB	BODB	DODB
**FTIR (wave number, cm** ^**−1**^ **)**			
Aromatic C–H	3062	3062.84	30 643.04
Aliphatic C–H	—	—	2855.46, 2938.14, 2962.67
C=O (coumarin)	1754.93	1754.90	1755.21
N=N (azo groups)	1511.39, 1565.82	1492.43, 1565.71	1459.38, 1566.51
*δ* lactone (O–C=O)	1204.67, 1039.57	1205.39, 1038.55	1205.10, 1039.25
C=N	1620.23	1620.96	1620.23
SO_3_H	3442.83	3443.43	3437.10

^**1**^ **H NMR (DMSO-*d***_**6**_ **), 400 MHz, *δ* (ppm)**			
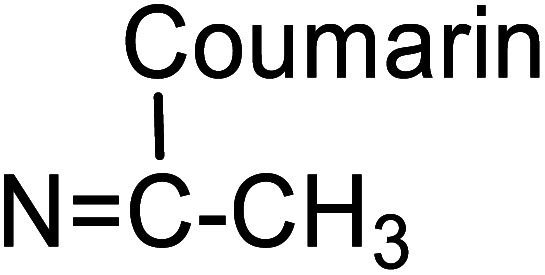	2.62 (6H, s)	2.61 (6H, s)	2.61 (6H, s)
Aromatic-H	7.07–8.83 (18H, m)	7.11–8.82 (22H, m)	7.07–8.83 (18H, m)
Coumarin-4-H	9.06, 9.25 (2H, s)	9.22, 9.26 (2H, s)	9.25, 9.29 (2H, s)
SO_3_H	10.36	10.37	10.32
CH_3_–O-Ar	—	—	3.03 (6H, s)

**Mass spectrum *m*/*z* (%)**			
(M^+.^)	816 (66.03%)	892 (43.42%)	952 (66.15%)
Molecular ion peak (base peak)	621 (100%)	724 (100%)	950 (100%)
Other peaks	734 (18.15%)	778 (93.95%)	404 (63.44%)
	341 (74.47%)		404 (15.70%)
	369 (26.45%)		363 (17.32%)
			257 (18.62%)
			177 (30.18%)

### 3.2. Electrochemical analysis

#### 3.2.1. Electrochemical frequency modulation measurements (EFM)

**Fig. 5:**
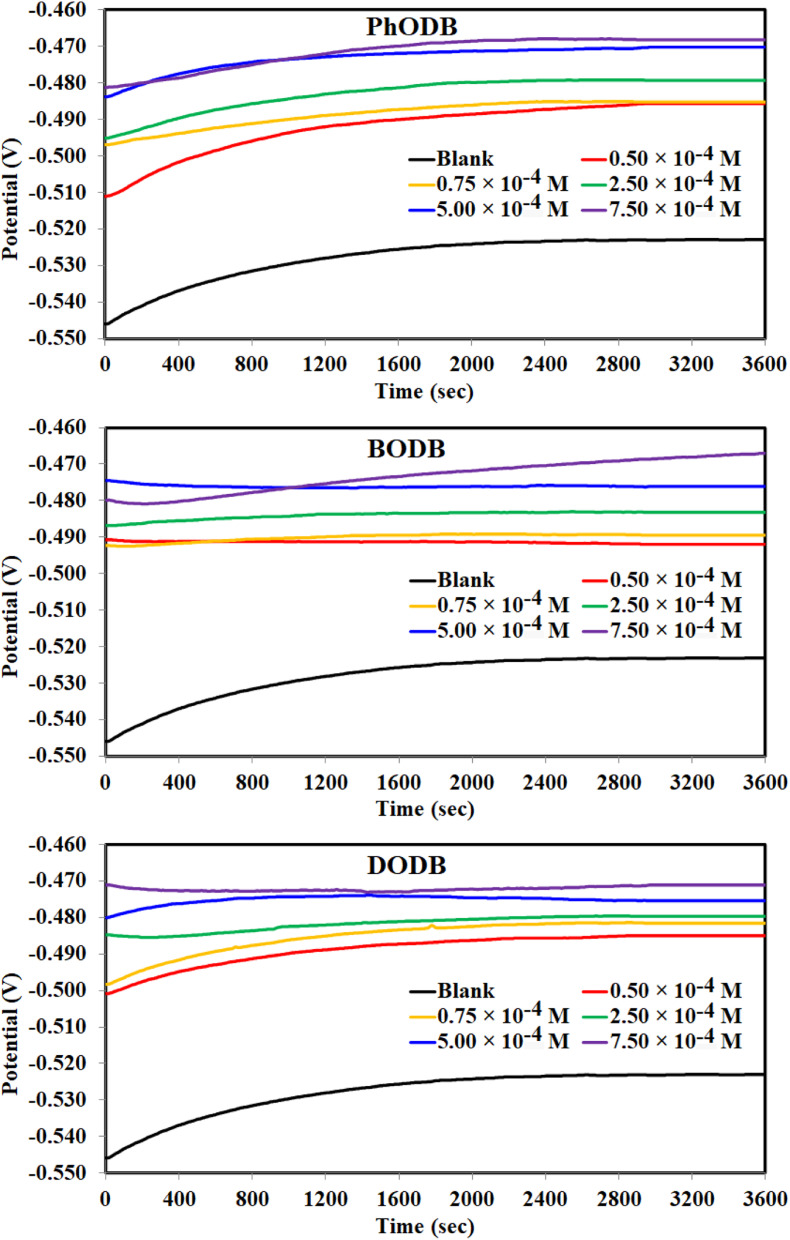
OCP curves for the corrosion of MS in 1.0 M HCl with and without different concentrations of synthesized coumarin derivatives (PhODB, BODB and DODB) at 25 °C.

**Fig. 6:**
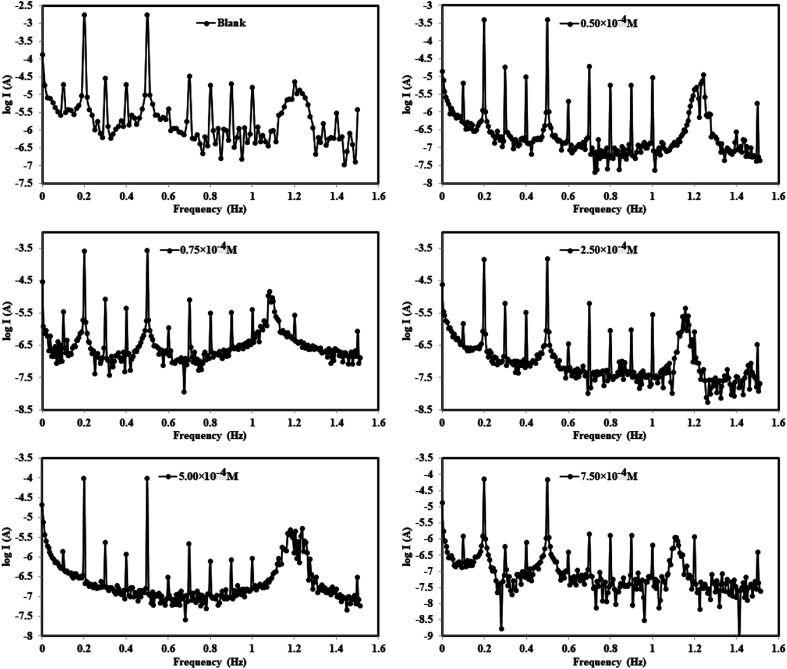
EFM curves for the corrosion of MS in 1.0 M HCl with and without different concentrations of synthesized inhibitor (BODB, for example) at 25 °C.

Before performing every electrochemical measurement for each concentration of the inhibitor, the working electrode (WE) was first immersed in acid electrolyte (1.0 M HCl) with and without inhibitors for 3600 s (1 h) to reach a stable steady state for the open circuit potential (OCP) (Fig. 5). EFM is a nondestructive measurement technology that uses a tiny electrical signal from an amphoteric current (AC) with varying frequencies to detect the rate of corrosion while producing two separate sine waves simultaneously. Intermodulation spectra at various concentrations for the DODB synthesized coumarin derivative are plotted in Fig. 6. Not only are the inputted frequencies included in the response of the current, but also their sums, multiplicities and differences.^55^ The frequency selection must be selective and tiny. The resulting data from EFM at higher peak can be used to determine the *i*_corr_ value without using the Tafel constants (*β*_a_ and *β*_c_). Causality factor values (CF-2 and CF-3) can be utilized to self-validate experimental results at various doses or concentrations. The recorded CF values are similar to the theoretical numbers (2 and 3) according to Table 2, and increasing the inhibitor dosages leads to a reduction in the current density values. By increasing the dosage of the coumarin derivative inhibitor, the severity of corrosion was reduced according to the estimated EFM values. According to the EFM results, the inhibition order is DODB > BODB > PhODB.

**Table 2:** Electrochemical kinetic parameters^a^ obtained by the EFM technique for MS in the absence and presence of various concentrations of PhODB, BODB and DODB inhibitors in 1.0 M HCl at 30 °C

Inhibitor name	Conc. (M)	*I*_corr_ (μA cm^−2^)		*β*_a_ (mV dec^−1^)	−*β*_c_ (mV dec^−1^)	CF-2	CF-3	*k* (mpy)	*θ*	*η*_EFM_%
Blank	—	2791	100.4		113.1	1.763	3.155	1275.00	—	—
PhODB	0.50 × 10^−4^	720.7	107.4		129.7	1.973	3.124	329.3	0.7418	74.18
	0.75 × 10^−4^	539.3	116.0		158.8	1.957	2.476	246.4	0.8068	80.68
	2.50 × 10^−4^	395.3	119.1		131.7	2.050	2.947	180.6	0.8584	85.84
	5.00 × 10^−4^	304.2	105.0		115.5	1.976	3.098	139.0	0.8910	89.10
	7.50 × 10^−4^	151.9	95.56		111.5	1.695	2.930	69.39	0.9456	94.56
BODB	0.50 × 10^−4^	653.7	100.6		124.5	1.994	3.256	298.70	0.7658	76.58
	0.75 × 10^−4^	485	125.2		132.3	2.040	3.121	221.60	0.8262	82.62
	2.50 × 10^−4^	347.2	105.0		112.8	2.308	3.023	158.7	0.8756	87.56
	5.00 × 10^−4^	215.9	97.59		101.8	2.574	3.387	98.66	0.9226	92.26
	7.50 × 10^−4^	114.9	100.8		120.0	1.994	3.100	52.52	0.9588	95.88
DODB	0.50 × 10^−4^	608.3	87.65		117.7	1.974	2.910	278.00	0.7820	78.20
	0.75 × 10^−4^	431.9	97.7		120.2	1.997	3.120	197.4	0.8453	84.53
	2.50 × 10^−4^	320	121.3		177.8	2.026	3.001	146.20	0.8853	88.53
	5.00 × 10^−4^	167.7	103.6		121.1	2.139	3.437	76.64	0.9399	93.99
	7.50 × 10^−4^	94.32	84.1		90.7	1.395	3.106	43.10	0.9662	96.62

a *E*_corr_ is the corrosion potential; *I*_corr_ is the corrosion current density: *β*_a_ and *β*_c_ are the Tafel constants for both anode and cathode; *k* is the corrosion rate; *θ* is the surface coverage; *η*_EFM_ is the inhibition efficiency.

#### 3.2.2. Electrochemical impedance spectroscopy (EIS) measurements

**Fig. 7:**
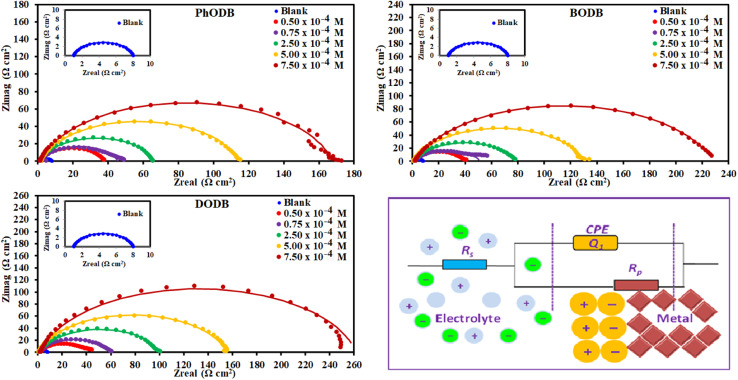
Nyquist plots for MS in 1.0 M HCl with and without different concentrations of synthesized coumarin derivatives (PhODB, BODB and DODB) at 25 °C and the equivalent circuit model for fitting the EIS data.

**Fig. 8:**
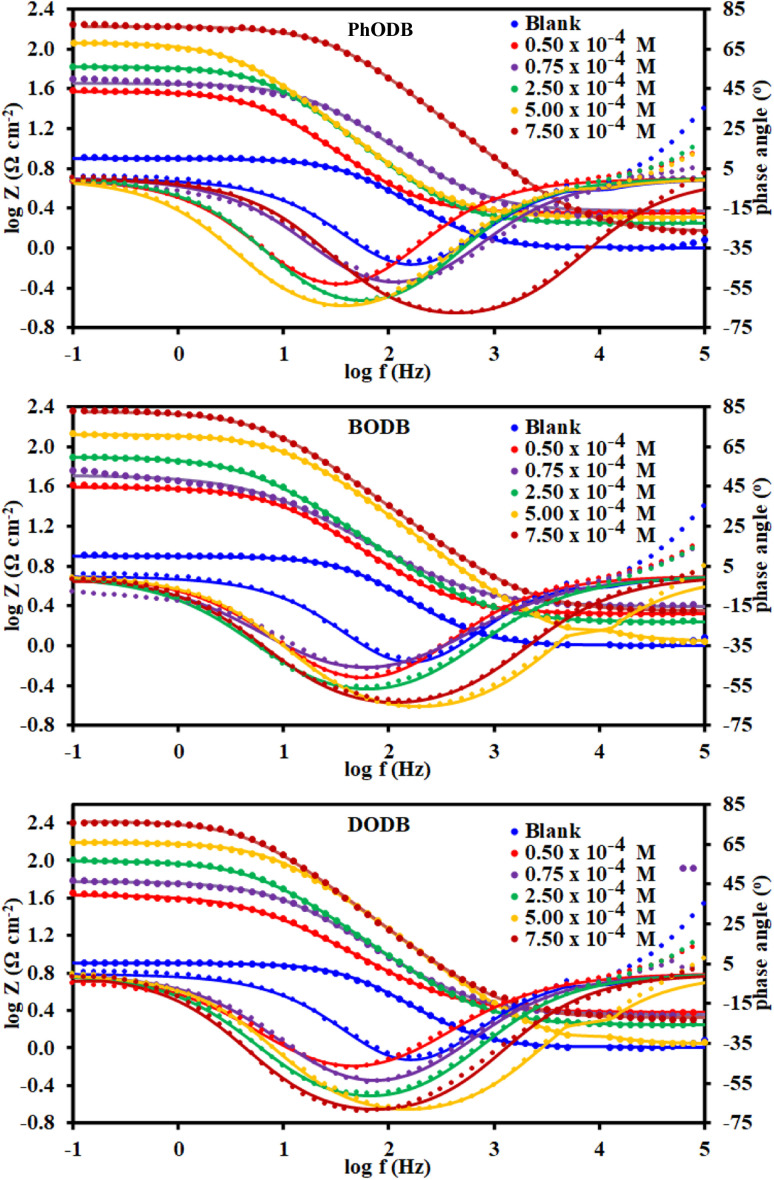
Bode and phase angle plots for steel in 1.0 M HCl with and without different concentrations of synthesized coumarin derivatives (PhODB, BODB and DODB) at 25 °C.

To study the interface characteristics and adsorption performance of the inhibitors, EIS is a very helpful measurement tool. EIS gives details of the kinetics and properties of electrochemical processes for a thorough knowledge of the corrosion inhibition process. Furthermore, EIS is another non-destructive method for analyzing a metal's corrosion inhibition performance in an acidic electrolyte. Using the EIS procedure, the inhibition characteristics of the three synthetic *p*-phenylenediamine and benzidine coumarin derivatives (PhODB, BODB and DODB) at different concentrations were identified at 25 °C. The comparable Nyquist plots and corresponding circuit model derived from EIS are displayed in Fig. 7. As can be seen from Fig. 7, a boost in inhibitor concentration forces the Nyquist plots to enlarge in diameter, which also leads their semi-circular shape to expand. As a result, the primary cause of the diameter increase is the formation of an inhibitor molecule film on the metal surface.^56^ For PhODB, BODB, and DODB at various concentrations, Fig. 7 illustrates the existence of a single capacitive loop, illuminating the activity of the examined inhibitors as major interface inhibitors and their adsorption onto the metal specimen surface. The Bode and phase angle plots for the investigated *p*-phenylenediamine and benzidine coumarin inhibitors (PhODB, BODB and DODB) are shown in Fig. 8. In order to analyze the electrode/electrolyte correlation by modeling the experimental graphs to plot the resulting data from EIS, a suitable circuit is necessary. To fit the Nyquist curve plots, many compartments are used to build up the equivalent circuit, electrolyte resistance (*R*_s_) with constant phase element (CPE) in a parallel combination together, and charge transfer resistance (*R*_ct_). Typically, CPE gives a suitable representation for the electrochemical process in place of capacitance.^57^ The CPE values can be represented by the following equation:

16 *Z*_CPE_ = (1/*Y*_0_)[j*ω*]^−*n*^

where, *Y*_0_ is CPE (constant), *n* is an exponent, j is the imaginary value and *ω* is the angular frequency. The measured *C*_dl_ values can be represented with the next equation:^58^

17 *C*_dl_ = (*Y*_0_*R*_ct_^1−*n*^)^1/*n*^

where, *Y*_0_ is CPE (constant) and *n* is the CPE exponent. Table 3 presents the measured values from EIS and it can be realized from these values that the *R*_ct_ values increase with a rise in inhibitor concentration, while *C*_dl_ demonstrates the opposite dependency. Nyquist and Bode graphs vary for the same inhibitor with different doses and also for the various investigated compounds. Even when using the same inhibitor, the strength of the highest peak increases with increasing concentration. Resistance may be indicated by the peak diameter, and resistance increases with increasing inhibitor concentration. Higher resistance (low *C*_dl_ values) might be amazing evidence for the presence of a layer from the evaluated inhibitors above the metal, providing excellent metal preservation and slowing down the corrosion rate.^59^ When inhibitors are present compared to when they are absent, the curve and loop diameter of the Nyquist plot are larger. With reference to the values in Table 3, the difference between *R*_ct_ and *R*_s_ values becomes greater with increasing inhibitor dosage and also with increasing *R*_ct_ values, and the *C*_dl_ values are lower due to inhibitor adsorption on the electrode surface being affected by increasing the thickness because of occupation of the electrode surface by inhibitors instead of water or acid electrolytes. The approximate values of *n* (close to unity) revealed that the electric double layer in the current investigation performed like a pseudo-capacitor type.^60,61^ As the concentration of PhODB, BODB, or DODB increases, the phase angle becomes wider due to the high frequency, as plotted in Fig. 8. By plotting log *Z* (Ω cm^2^) against log frequency (Hz), the capacitance properties of the adsorption behavior of the investigated compounds on the MS surface were confirmed.^62^ The observed values from EIS indicate that, at higher inhibitor dosage, the highest mitigation results can be achieved. According to the EIS results, the inhibition order is DODB > BODB > PhODB.

**Table 3:** EIS parameters for corrosion of MS in 1.0 M HCl in the absence and presence of different concentrations of PhODB, BODB and DODB inhibitors at 25 °C^a^

Inhibitor	Conc. (M)	*R*_s_ (*R*_u_) (Ω cm^2^)	*R*_ct_ (*R*_p_) (Ω cm^2^)	*Y*_0_ (μΩ^−1^ s^*n*^ cm^−2^)	*n*	*C*_dl_ (μF cm^−2^)	*χ* ^2^ (chi squared)	*S*	*α*°	*τ* (mS)	*θ*	*η*_z_%
Blank	—	1.082	6.884	478.50	0.8836	225.363	2.25 × 10^−2^	−0.365	−42.07	3.29	—	—
PhODB	0.50 × 10^−4^	2.265	35.55	1063.00	0.8760	668.585	3.33 × 10^−3^	−0.481	−53.43	37.79	0.8064	80.64
	0.75 × 10^−4^	2.368	43.20	395.60	0.8434	185.830	3.83 × 10^−2^	−0.588	−52.62	17.09	0.8406	84.06
	2.50 × 10^−4^	1.785	63.88	532.10	0.8833	340.376	5.06 × 10^−3^	−0.661	−61.75	33.99	0.8922	89.22
	5.00 × 10^−4^	2.029	112.1	317.40	0.8708	193.487	3.34 × 10^−3^	−0.646	−64.00	35.58	0.9386	93.86
	7.50 × 10^−4^	1.458	166.4	68.48	0.8637	33.798	9.11 × 10^−4^	−0.678	−67.71	11.40	0.9586	95.86
BODB	0.50 × 10^−4^	2.067	37.56	886.40	0.8273	435.682	5.12 × 10^−3^	−0.545	−51.52	33.29	0.8167	81.67
	0.75 × 10^−4^	2.440	49.30	1176.00	0.738	427.903	7.34 × 10^−3^	−0.513	−46.87	57.98	0.8604	86.04
	2.50 × 10^−4^	1.729	77.18	738.60	0.8004	361.547	4.73 × 10^−3^	−0.620	−56.11	57.01	0.9108	91.08
	5.00 × 10^−4^	1.086	129.6	121.00	0.8390	54.512	1.59 × 10^−3^	−0.741	−65.86	15.68	0.9469	94.69
	7.50 × 10^−4^	2.206	225.9	704.70	0.8201	470.907	5.07 × 10^−4^	−0.721	−62.86	159.19	0.9695	96.95
DODB	0.50 × 10^−4^	2.360	40.69	1354.00	0.766	558.5319	4.83 × 10^−3^	−0.482	−47.66	55.09	0.8308	83.08
	0.75 × 10^−4^	2.252	57.06	587.60	0.818	276.055	7.74 × 10^−2^	−0.598	−54.43	33.53	0.8794	87.94
	2.50 × 10^−4^	1.762	98.15	494.40	0.8373	274.626	5.42 × 10^−3^	−0.676	−60.41	48.53	0.9299	92.99
	5.00 × 10^−4^	1.093	154.0	116.10	0.8618	60.894	2.11 × 10^−3^	−0.775	−68.15	17.88	0.9553	95.53
	7.50 × 10^−4^	2.106	260.0	1733.00	0.8667	1533.021	1.96 × 10^−3^	−0.771	−68.92	450.58	0.9735	97.35

a *R*_s_ = solution resistance, *R*_ct_ = charge transfer resistance, Y_0_, *n* = constant phase elements, *C*_dl_ = double layer capacitance, *S* = the slopes of the Bode impedance magnitude at intermediate frequencies, *α*° = maximum phase angle, □ = the relaxation time, *θ* = surface coverage, *η*_z_ = inhibition efficiency.

#### 3.2.3. Potentiodynamic polarization measurements (PDP)

**Fig. 9:**
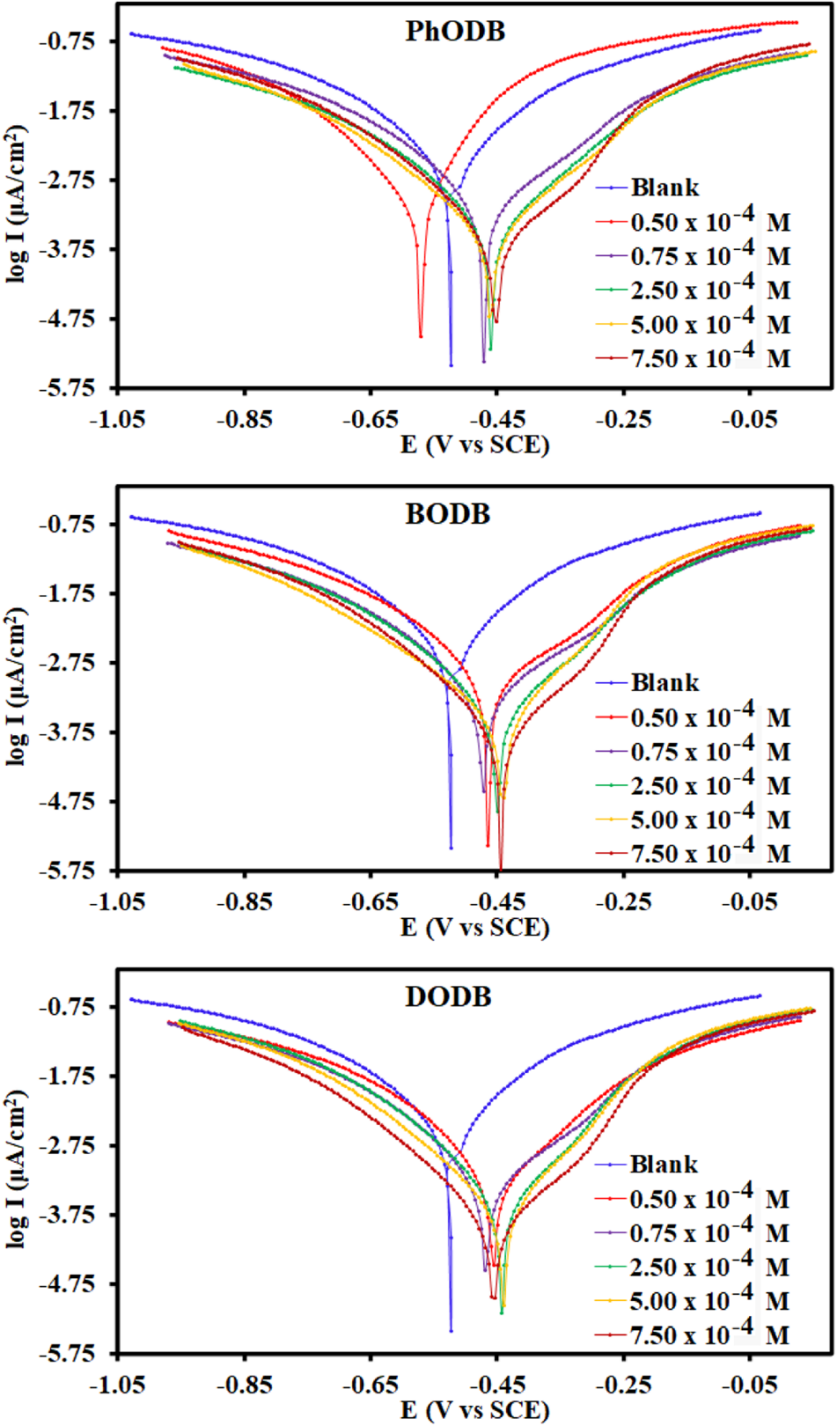
PDP curves for the corrosion of steel in 1.0 M HCl with and without different concentrations of synthesized coumarin derivatives (PhODB, BODB and DODB) at 25 °C.

In the current study, PDP measurements were used to evaluate the adsorptive capability of synthesized coumarin derivatives *i.e.*, PhODB, BODB and DODB. The PDP method is applied to explain the interaction between the electrical charge and the electrode potential. PDP analysis was performed using various dosages of inhibitors (PhODB, BODB and DODB) at 25 °C (Fig. 9). Cathodic (*β*_c_), and anodic (*β*_a_) Tafel slopes, current density (*i*_corr_), corrosion potential (*E*_corr_), degree of surface coverage (*θ*) and inhibition efficiency (*η*_PDP_%) values were obtained and are listed in Table 4. Using inhibitors and as a result of blocking the active points on the electrode surface due to formation of a protective layer, inhibition efficiency (*η*_PDP_%) increased, and corrosion rate and current density values were reduced. No significant or valuable shift were realized in the *E*_corr_ results, just a tiny shift to the positive side direction. As recorded in other studies,^63–65^ if *E*_corr_ values are higher than 85 mV, the inhibitor can be anodic or cathodic according to the *E*_corr_ values recorded for the acidic electrolyte. Furthermore, inhibitors with values of *E*_corr_ lower than 85 mV are confirmed to be mixed type (anodic and cathodic types together). From the *E*_corr_ results in Table 4, the PhODB, BODB, and DODB inhibitors are also considered to be mixed-type inhibitors, due to the slight changes in *β*_c_ and *β*_a_. The Tafel lines are parallel, indicating that there was no change in the mechanism of the process in the presence and absence of inhibitors. As the *θ* values increased, *i*_corr_ values reduced as a result of an anodic protective film formed on the electrode due to the presence of coumarin inhibitors. According to the PDP results, the inhibition order is DODB > BODB > PhODB.

**Table 4:** Electrochemical parameters^a^ for steel dissolution in 1.0 M HCl solution containing different concentrations of the PhODB, BODB and DODB inhibitors obtained from polarization measurements at 25 °C

Inhibitor name	Conc. (M)	*E*_corr_ *vs.*. SCE (mV)	*I*_corr_ (μA cm^−2^)	*β*_a_ (mV dec^−1^)	−*β*_c_ (mV dec^−1^)	*k* (mpy)	Δ*E*_corr_ (mV)	*θ*	*η*_PDP_%
Blank	—	−523	8624	305.0	333.0	4137	—	—	—
PhODB	0.50 × 10^−4^	−571	2340	246.9	212.6	1234	−48	0.729	72.85
	0.75 × 10^−4^	−471	1350	233.7	240.8	617.9	52	0.843	84.34
	2.50 × 10^−4^	−459	778	211.8	230.7	355.5	64	0.910	90.97
	5.00 × 10^−4^	−460	580	199.9	210.3	265.2	63	0.933	93.27
	7.50 × 10^−4^	−452	406	175.7	192.6	185.5	71	0.953	95.29
BODB	0.50 × 10^−4^	−465	1440	220.7	232.0	659.0	58	0.833	83.29
	0.75 × 10^−4^	−472	1050	226.8	233.0	480.1	51	0.878	87.82
	2.50 × 10^−4^	−447	652	191.8	207.7	297.8	76	0.924	92.44
	5.00 × 10^−4^	−441	478	175.0	213.0	218.5	82	0.945	94.45
	7.50 × 10^−4^	−444	302	166.7	187.1	138.0	79	0.965	96.50
DODB	0.50 × 10^−4^	−465	1370	234.4	246.1	624.9	58	0.841	84.11
	0.75 × 10^−4^	−468	937	215.3	224.3	427.9	55	0.891	89.13
	2.50 × 10^−4^	−443	569	182.0	203.8	260.1	80	0.934	93.40
	5.00 × 10^−4^	−440	419	169.8	197.7	191.2	83	0.951	95.14
	7.50 × 10^−4^	−455	207	163.1	174	94.73	68	0.976	97.60

a *E*_corr_ is the corrosion potential; *I*_corr_ is the corrosion current density: *β*_a_ and *β*_c_ are Tafel constants for both anode and cathode; *k* is the corrosion rate; *θ* is the surface coverage; *η*_PDP_ is the inhibition efficiency.

### 3.3. Gravimetric measurements (weight loss)

The WL gravimetric measurement technique is a successful and frequently utilized procedure that does not need a well-established research facility to execute in order to investigate the actual character of organic inhibitors. The effect of different dosages of PhODB, BODB and DODB inhibitors on MS in corrosive electrolyte was investigated and the same behavior was also investigated at various temperatures.

#### 3.3.1. Effect of different concentrations

**Fig. 10:**
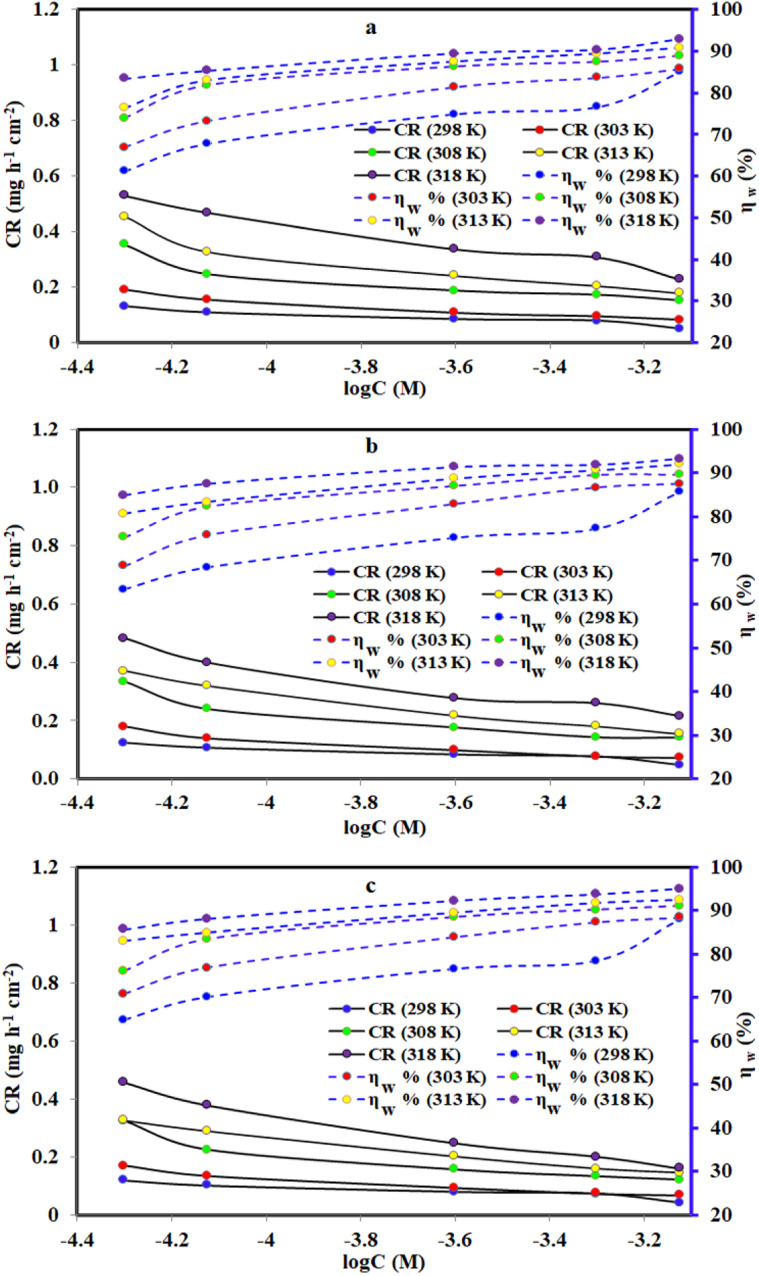
Effect of various temperatures and concentrations of synthesized coumarin derivatives: (a) PhODB, (b) BODB and (c) DODB on the corrosion rate of steel in 1.0 M HCl using the weight loss method.

Using various dosages of PhODB, BODB, and DODB inhibitors, the corrosion rate for MS in (1.0 M HCl) corrosive electrolyte was investigated. The MS electrode was submerged in the corrosive electrolyte for 24 h and the weight was determined before and after submersion. The effect of adding five different concentrations of PhODB, BODB, and DODB inhibitors on the corrosion rate was investigated. Furthermore, the same effect was studied at various temperatures (298, 303, 308, 313 and 318 K). Several variables like corrosion rate (CR(*k*) = mg cm^−2^ h^−1^), surface coverage (*θ*) and inhibition efficiency (*η*_WL_%) were measured and are listed in Table 5. From the values presented in Table 5, as the dosage of the inhibitors is raised, the *η*_WL_% values improve and the CR values reduce. At 318 K, the *η*_WL_% values are 95.85%, 93.19% and 94.98% at the highest concentration (7.5 × 10^−4^ M) for the studied inhibitors PhODB, BODB, and DODB, respectively. At the same mentioned temperature and without adding inhibitors, the CR value was 03.1736 mg cm^−2^ h^−1^ and after adding inhibitors (7.5 × 10^−4^ molar concentration) the CR values are reduced to become 0.2269 mg cm^−2^ h^−1^, 0.2162 mg cm^−2^ h^−1^ and 0.1592 mg cm^−2^ h^−1^ for PhODB, BODB, and DODB, respectively. From the indicated values in Table 5 and Fig. 10, the *η*_WL_% results increase with increases in both temperatures and concentrations of PhODB, BODB and DODB inhibitors, indicating chemical adsorption. According to the WL results, the inhibition order is DODB > BODB > PhODB.

**Table 5:** Corrosion rate, surface coverage and percentage of inhibition efficiency of steel in 1.0 HCl of the PhODB, BODB and DODB inhibitors at different temperatures

Inhibitor	Inhibitor conc. (M)	25 °C			30 °C			35 °C			40 °C			45 °C		
		*C*_R_ (*k*) (mg cm^−2^ h^−1^)	*θ*	*η*_w_ (%)	*C*_R_ (*k*) (mg cm^−2^ h^−1^)	*θ*	*η*_w_ (%)	*C*_R_ (*k*) (mg cm^−2^ h^−1^)	*θ*	*η*_w_ (%)	*C*_R_ (*k*) (mg cm^−2^ h^−1^)	*θ*	*η*_w_ (%)	*C*_R_ (*k*) (mg cm^−2^ h^−1^)	*θ*	*η*_w_ (%)
Blank	0.00 × 10^−4^	0.3365	—	—	0.5765	—	—	1.3534	—	—	1.9141	—	—	3.1736	—	—
PhODB	0.50 × 10^−4^	0.1309	0.611	61.11	0.1913	0.668	66.82	0.3541	0.738	73.84	0.4537	0.763	76.30	0.5290	0.833	83.33
	0.75 × 10^−4^	0.1084	0.678	67.78	0.1547	0.732	73.17	0.2472	0.817	81.74	0.3264	0.830	82.95	0.4680	0.853	85.25
	2.50 × 10^−4^	0.0846	0.749	74.87	0.1075	0.814	81.35	0.1870	0.862	86.18	0.2407	0.874	87.42	0.3366	0.894	89.39
	5.00 × 10^−4^	0.0788	0.766	76.58	0.0947	0.836	83.57	0.1717	0.873	87.32	0.2041	0.893	89.33	0.3074	0.903	90.31
	7.50 × 10^−4^	0.0505	0.850	85.00	0.0824	0.857	85.71	0.1514	0.888	88.81	0.1780	0.907	90.70	0.2269	0.929	92.85
BODB	0.50 × 10^−4^	0.1233	0.634	63.38	0.1801	0.688	68.75	0.3346	0.753	75.28	0.3706	0.806	80.64	0.4815	0.848	84.83
	0.75 × 10^−4^	0.1063	0.684	68.40	0.1396	0.758	75.79	0.2399	0.823	82.27	0.3192	0.833	83.32	0.3986	0.874	87.44
	2.50 × 10^−4^	0.0835	0.752	75.17	0.0990	0.828	82.83	0.1762	0.870	86.98	0.2170	0.887	88.66	0.2773	0.913	91.26
	5.00 × 10^−4^	0.0766	0.772	77.23	0.0772	0.866	86.60	0.1424	0.895	89.48	0.1804	0.906	90.58	0.2600	0.918	91.81
	7.50 × 10^−4^	0.0485	0.856	85.58	0.0723	0.875	87.46	0.1415	0.895	89.55	0.1542	0.919	91.94	0.2162	0.932	93.19
DODB	0.50 × 10^−4^	0.1184	0.648	64.82	0.1685	0.708	70.77	0.3240	0.761	76.06	0.3263	0.830	82.96	0.4566	0.856	85.61
	0.75 × 10^−4^	0.1007	0.701	70.08	0.1335	0.768	76.85	0.2247	0.834	83.40	0.2895	0.849	84.87	0.3780	0.881	88.09
	2.50 × 10^−4^	0.0789	0.766	76.56	0.0930	0.839	83.87	0.1563	0.884	88.45	0.2013	0.895	89.48	0.2471	0.922	92.21
	5.00 × 10^−4^	0.0725	0.784	78.44	0.0734	0.873	87.27	0.1330	0.902	90.17	0.1588	0.917	91.71	0.2004	0.937	93.69
	7.50 × 10^−4^	0.0407	0.879	87.90	0.0671	0.884	88.36	0.1212	0.910	91.04	0.1445	0.925	92.45	0.1592	0.950	94.98

#### 3.3.2. Adsorption isotherm

**Fig. 11:**
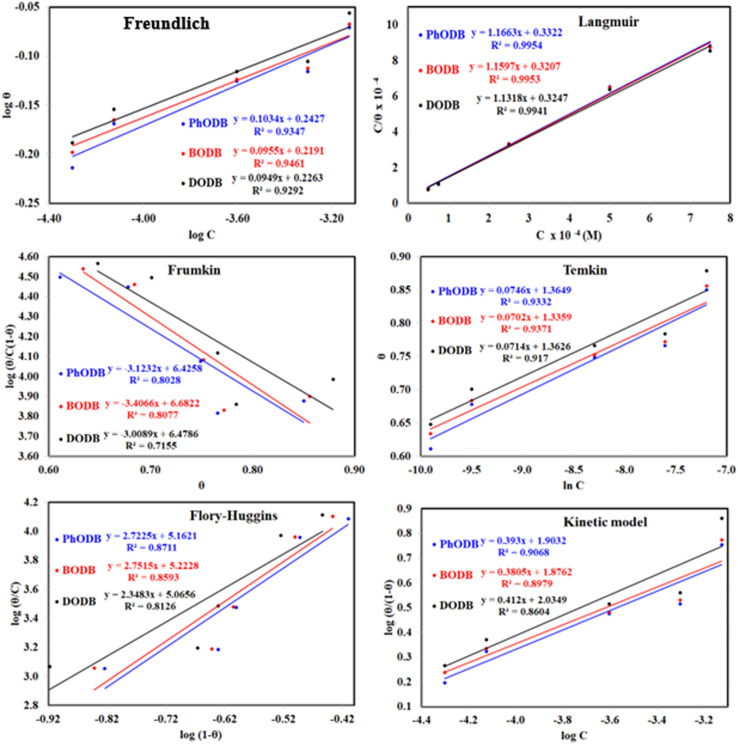
Different adsorption isotherms for synthesized coumarin derivatives (PhODB, BODB and DODB) using the weight loss method.

Understanding how the inhibitors and active points on the metal electrode surface interact is the main goal of the adsorption isotherm. In the current investigation, a variety of isotherms were utilized for fitting, with the Langmuir model providing the best match since the linear regression coefficients (*R*^2^) are nearly all equal to one.^66^ The *R*^2^ values for Freundlich, Langmuir, Frumkin, Temkin, Flory–Huggins and kinetic–thermodynamic adsorption isotherms models are listed and plotted in Table 6 and Fig. 11, respectively. The next formula is used to describe the Langmuir adsorption isotherm:^67^

18 *C*/*θ* = 1/*K*_ads_ + *C*

where, *C* = inhibitor concentration, *θ* = surface coverage and *K* = binding constant. By plotting *C vs.* (*C*/*θ*), straight lines were achieved for the Langmuir model (Fig. 11). Considering the intercept, the Gibb's standard free energy 
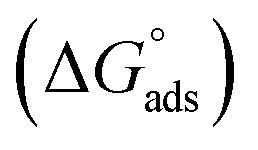
 can be calculated from the following formula:^68^19
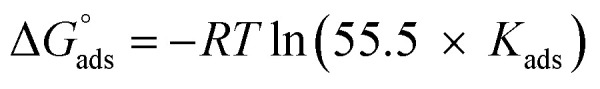
where, *R* is the universal gas constant (8.314 J mol^−1^ K^−1^), *T* is the temperature (kelvin) and the numerical value (55.5 mole per liter) is the water concentration. The calculated 
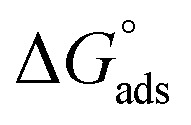
 and K_ads_ values for all adsorption isotherm models are listed in Table 6 at 298 K. Spontaneous nature of adsorption and stability of the adsorbed layer are expected for the studied organic derivatives towards the electrode metal surface due to the negative calculated values for 
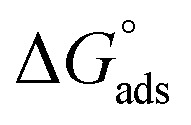
. Based on previously accepted research,^69,70^ the spontaneous adsorption behavior is a steady process that cannot be reversed. The adsorption process behavior depends on the 
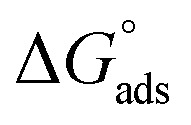
 values: physisorption if 
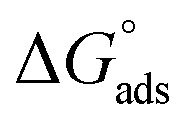
 ≥ −20 kJ mol^−1^, chemisorption if 
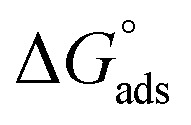
 ≥ −40 kJ mol^−1^ and mixed type if 
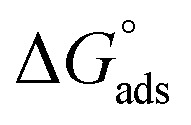
 values are between −20 and −40 kJ mol^−1^.^71^ The 
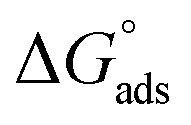
 results are −36.10, −36.18 and 36.15 kJ mol^−1^ for PhODB, BODB and DODB, respectively, according to the Langmuir adsorption model, so the adsorption of these compounds on MS surfaces are of mixed type (physisorption and chemisorption, but mainly chemical).

**Table 6:** Adsorption isotherm models of the inhibitors with values of *R*^2^, slopes, intercepts, *K*_ads_ and 
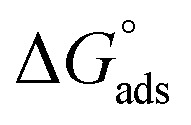
 obtained by using data from WL measurements^a^

Adsorption isotherm model	Linear form equation	Inhibitor	Slope	Intercept	*R* ^2^	*K*_ads_, M^−1^	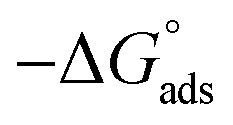 , kJ mol^−1^
Freundlich	log *θ* = log *K* + *1/n* log *C*	PhODB	0.10341	0.24274	0.93471	1.7488	11.53
		BODB	0.09548	0.21909	0.94605	1.6561	11.39
		DODB	0.09492	0.22631	0.92921	1.6839	11.43
Langmuir	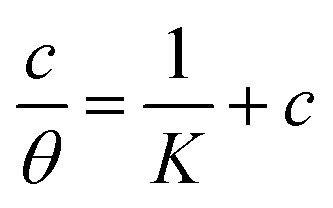	PhODB	1.16630	0.00003	0.99535	30 105	36.10
		BODB	1.15966	0.00003	0.99529	31 183	36.18
		DODB	1.13180	0.00003	0.99408	30 800	36.15
Frumkin	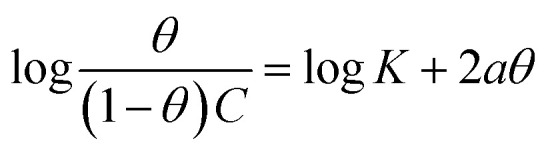	PhODB	−3.12323	6.42578	0.80282	2.6655 × 10^6^	47.39
		BODB	−3.40660	6.68223	0.80769	4.8109 × 10^6^	48.88
		DODB	−3.00893	6.47858	0.71547	3.0101 × 10^6^	47.70
Temkin	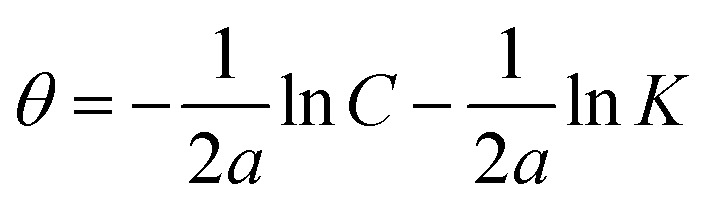	PhODB	12.50756	−17.63890	0.93319	0.2441	6.57
		BODB	13.35511	−18.37582	0.93713	0.2526	6.65
		DODB	12.83880	−18.19938	0.91703	0.2423	6.55
Flory–Huggins	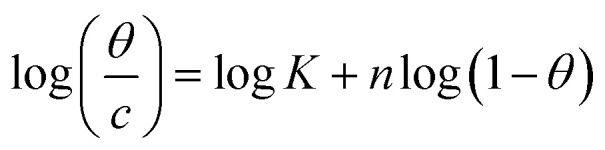	PhODB	2.72247	5.16207	0.87112	1.4523 × 10^5^	40.06
		BODB	2.75152	5.22275	0.85930	1.6701 × 10^5^	40.41
		DODB	2.34830	5.06561	0.81262	1.1631 × 10^5^	39.50
Kinetic–thermodynamic	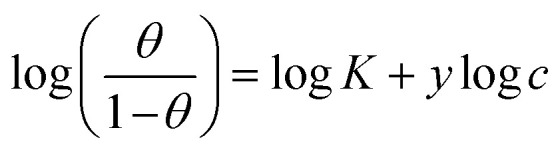	PhODB	0.39304	1.90316	0.90679	80.0135	21.16
		BODB	0.38050	1.87625	0.89787	75.2056	21.00
		DODB	0.41203	2.03494	0.86044	108.3769	21.92

a *R* ^2^ = regression correlation coefficient, *K* = binding constant, *θ* = surface coverage, *c* = concentration.

According to the Van't Hoff equation, the adsorption thermodynamic parameters for the synthesized inhibitors (PhODB, BODB and DODB) on the MS electrode surface are essential for understanding the adsorption process and this equation can be represented as follows:^72,73^20
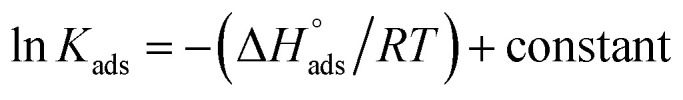


By fitting (1/*T*) *vs.* (*K*_ads_), the adsorption heat value 
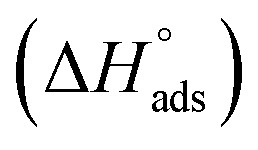
 can be retrieved as a result of the slope 
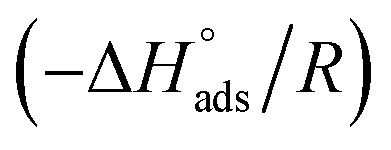
. The 
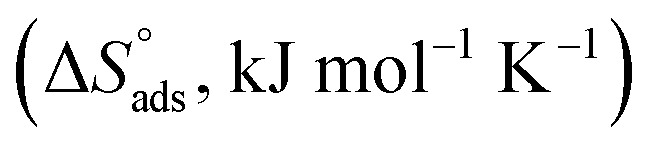
 standard adsorption entropy can retrieved from the following basic thermodynamic equation:^74^21
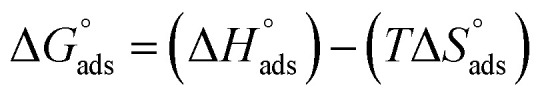


The values of the adsorption parameters are depicted in Table 7. The adsorption mechanism can be identified according to the resulting 
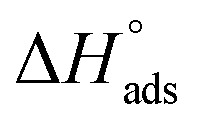
 values: if the values are negative, it is an exothermic physisorption or chemisorption mechanism; and if the values are positive, it is an endothermic or chemisorption mechanism.^75^ The values were +ve and between 48.77 and 56.00 kJ mol^−1^. These values are more than 41.8 kJ mol^−1^, indicating that the adsorption is chemical and the +ve sign indicates that the adsorption process is endothermic, *i.e.* chemisorption. The values of 
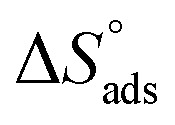
 are positive, indicating that the increase in disorderly due to the replacement of water molecules from the MS surface.

**Table 7:** Adsorption parameters obtained from the Langmuir isotherm for steel dissolution in 1.0 M HCl in the presence of PhODB, BODB and DODB inhibitors at different temperatures

Inhibitor	Temp. (K)	*K*_ads_ (kJ mol^−1^)	Δ*G*_ads_ (kJ mol^−1^)	Δ*S*_ads_ (J mol^−1^ K^−1^)	Δ*H*_ads_ (kJ mol^−1^)
PhODB	298	30 105	−36.10	282.78	48.77
	303	53 526	−37.55	284.87	
	308	91 061	−39.53	286.68	
	313	89 836	−40.13	284.04	
	318	107 920	−41.26	283.11	
BODB	298	31 183	−36.18	317.20	58.94
	303	58 072	−37.75	319.10	
	308	98 126	−39.72	320.31	
	313	99 071	−40.39	317.33	
	318	153 898	−42.20	318.03	
DODB	298	30 800	36.15	307.23	56.00
	303	61 915	−37.91	309.95	
	308	95 196	−39.64	310.52	
	313	117 823	−40.84	309.39	
	318	130 415	−41.76	307.42	

#### 3.3.3. Thermodynamic and activation parameters

**Fig. 12:**
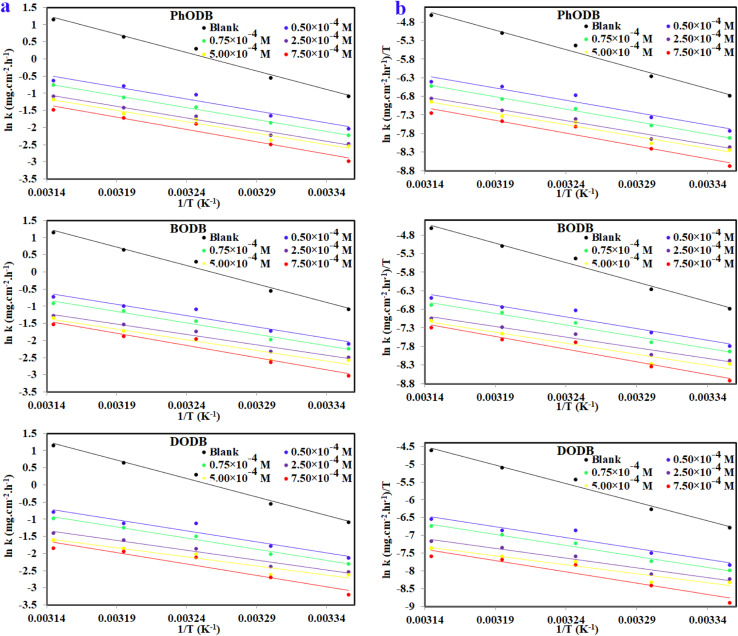
Arrhenius plots (a) and transition state plots (b) for steel dissolution with and without various dosages from synthesized coumarin derivatives (PhODB, BODB and DODB) in 1.0 M HCl solution.

Monitoring the corrosion behavior at various temperatures is very important to produce various related activation (energy 
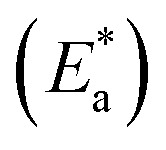
, entropy (ΔS*) and enthalpy (ΔH*)) parameters, as listed in Table 8. The correlation between temperature (*T*) and corrosion rate (*k*) is typically represented by the Arrhenius equation^76^ as follows:

22 log CR(*k*) = −log *A* − (*E*_a_/2.303*RT*)

where, *A* is a frequency factor, *E*_a_ is the activation energy, *R* is the molar gas constant (8.314 J mol^−1^ K^−1^) and *T* is the absolute temperature in K. Fig. 12(a) shows the plots for the Arrhenius relation between (1/*T*) and log corrosion rate (*k* = mg cm^−2^ h^−1^) for PhODB, BODB and DODB inhibitors. From Table 8, 
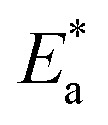
 = 89.76 kJ mol^−1^ for an uninhibited corrosive electrolyte and the values changed to 59.72, 59.21 and 55.37 kJ mol^−1^ for PhODB, BODB and DODB, respectively, at 7.50 × 10^−4^ molar concentration for all organic inhibitors. This change in 
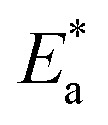
 numbers is a result of the chemisorption adsorption behavior of these inhibitors. The transition state equation can be represented as in the next equation:^77^23
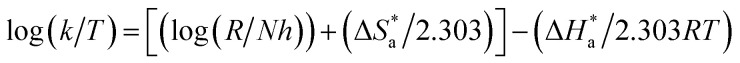
where, 
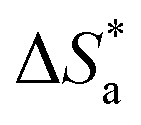
 and 
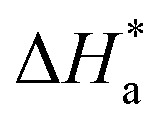
 are the activation entropy and enthalpy, respectively. *N* is Avogadro's number (6.022 × 10^23^ mol^−1^), *h* is Planck's constant (6.626176 × 10^−34^ J s) and *T* is the temperature in kelvin (K). Fig. 12(b) shows plots of the transition state relation between (1/*T*) and log(*k*/*T*). The DODB compound has the lowest activation value among the investigated synthesized organic derivatives and because of this, it is suggested that it would be a more effective inhibitor for MS against an acidic electrolyte. According to thermodynamic and activation parameter results, the inhibition order is DODB > BODB > PhODB.

**Table 8:** Activation parameters values for steel in 1.0 M HCl in the absence and presence of different concentrations of the PhODB, BODB and DODB compounds

Inhibitor	Conc. of inhibitor (M)	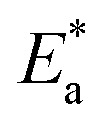 (kJ mol^−1^)	ΔH* (kJ mol^−1^)	ΔS* (J mol^−1^ K^−1^)
Blank	0.00 × 10^−4^	89.76	87.20	38.86
PhODB	0.50 × 10^−4^	57.82	55.26	−75.91
	0.75 × 10^−4^	57.89	55.34	−77.67
	2.50 × 10^−4^	56.24	53.68	−85.59
	5.00 × 10^−4^	54.98	52.42	−90.57
	7.50 × 10^−4^	59.72	57.16	−77.09
BODB	0.50 × 10^−4^	54.48	51.92	−87.52
	0.75 × 10^−4^	54.76	52.20	−88.40
	2.50 × 10^−4^	50.24	47.68	−105.84
	5.00 × 10^−4^	51.79	49.23	−102.12
	7.50 × 10^−4^	59.21	56.65	−79.46
DODB	0.50 × 10^−4^	53.11	50.55	−92.50
	0.75 × 10^−4^	53.95	51.39	−91.57
	2.50 × 10^−4^	48.18	45.62	−113.22
	5.00 × 10^−4^	44.18	41.63	−127.75
	7.50 × 10^−4^	55.37	52.81	−93.21

### 3.4. Spectral UV-visible analysis

**Fig. 13:**
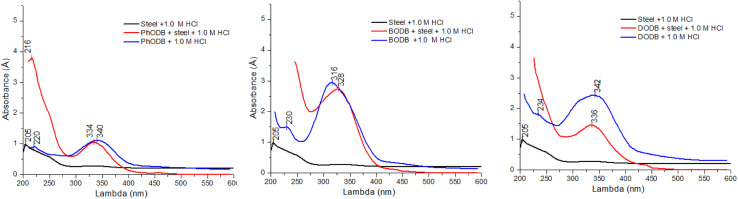
UV-visible spectra for various solutions for steel, 1.0 M HCl and PhODB, BODB and DODB inhibitors at 25 °C after immersion for 24 h.

Applying UV-visible spectroscopic analysis, one can ascertain how the coumarin derivatives and metallic cations form a complex. The MS electrode was exposed to (1.0 M HCl) corrosive electrolyte for 24 h at 25 °C without using any inhibitor (blank = MS + 1.0 M HCl). Also, a significant molar concentration (1.25 × 10^−4^) of each coumarin derivative inhibitor was dissolved in the same corrosive electrolyte to be used for the blank sample (solution A = inhibitor + 1.0 M HCl). Furthermore, another solution contained an MS electrode and defined significant molar dosages of the inhibitors (1.25 × 10^−4^) dissolved and immersed again in the same corrosive electrolyte (1.0 M HCl) for 24 h at 25 °C (solution B = inhibitor + MS + 1.0 M HCl). The UV was measured for the three different solutions for each coumarin inhibitor and the absorption wavelengths were recorded, as plotted in Fig. 13. The measured absorption wavelength for the blank (MS + 1.0 M HCl) solution was 205 nm. For solution A (inhibitor + 1.0 M HCl), the absorption values were 220 nm, 230 nm and 234 nm for PhODB, BODB and DODB, respectively, as a result of π–π* transitions. Also, other values were obtained for the same solutions at 334 nm, 316 nm and 342 nm for PhODB, BODB and DODB, respectively, as a result of n–π* transitions (hypsochromic shift). Furthermore, for solution B (inhibitor + MS + 1.0 M HCl), the measured absorption values were 340 nm, 328 nm and 336 nm for PhODB, BODB and DODB, respectively, as a result of π–π* transitions (bathochromic shift). This variation in absorption data might be interpreted as a sign that the PhODB, BODB and DODB coumarin inhibitors and the metallic electrode surface are forming a complex.^78^

### 3.5. SEM and EDX

**Fig. 14:**
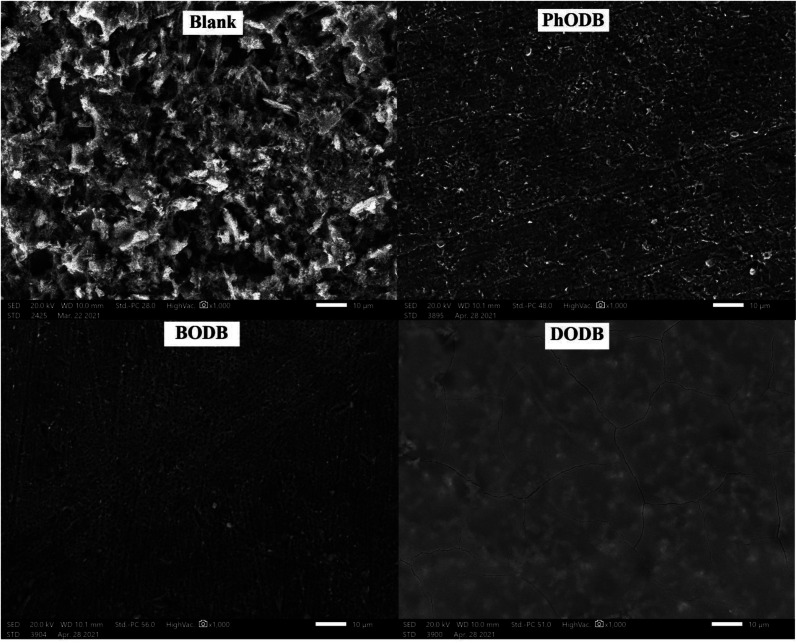
SEM images for steel surface after immersion in 1.0 M HCl for 24 h in the absence and presence of synthesized coumarin derivatives (PhODB, BODB and DODB) at 25 °C.

**Fig. 15:**
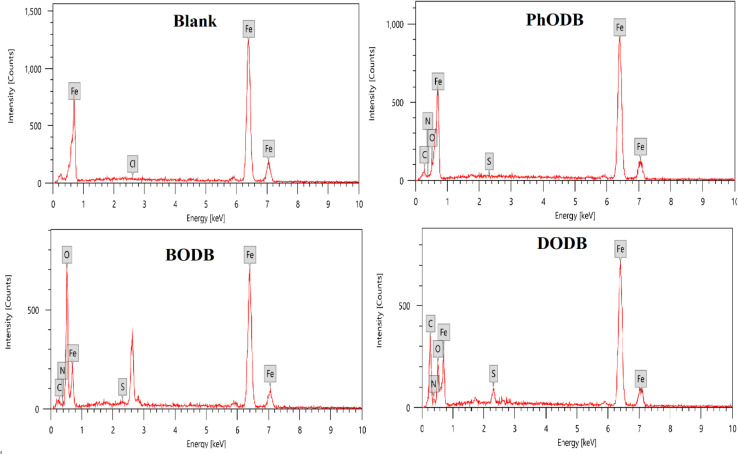
EDX spectra for steel surface after immersion in 1.0 M HCl for 24 h in the absence and presence of synthesized coumarin derivatives (PhODB, BODB and DODB) at 25 °C.

In order to validate the electrochemical measurements, quantitative EDX studies and qualitative microscopic SEM analyses were carried out. The surface states of the MS samples are shown in Fig. 14 before and after 24 hours of immersion in 1 M HCl solution and in the presence of (7.5 × 10^−4^) PhODB, BODB and DODB inhibitors. Without inhibitors, the sample has been substantially degraded by the medium and has become heterogeneous as a result of the acid's aggressive attack (Fig. 15(a)). The MS surface is noticeably enhanced, has fewer holes, is smoother, and is more heterogeneous in the presence of the PhODB, BODB and DODB inhibitors at 7.5 × 10^−4^ M, as seen in Fig. 14, which confirms the inhibitory effect. The inhibitors acted as an isolation layer deposited on and protecting the MS surface from Cl^−^ ion attack. To identify the composition of the elements deposited on the MS surface, EDX measurements were applied. The obtained EDX results are plotted in Fig. 15 and the percentages of detected ions are listed in Table 9. For the blank sample (no inhibitor added), the major detected ions are mainly Fe (99.94 wt%) and Cl (0.06 wt%). In the case of adding PhODB, BODB and DODB inhibitors, new sufficient concentrations were detected for new elements (C, S, N and O) and corrosive chloride ions were absent. This behavior may suggest that the synthesized organic compounds have high adsorption characteristics for deposition on MS and a preventative film from the inhibitors has formed on the metal surface.^79,80^

**Table 9:** EDX analysis for steel surface after 24 h immersion in 1.0 M HCl in the presence and absence of the synthesized inhibitors PhODB, BODB and DODB

Element	Blank (HCl–Fe)		PhODB-Fe		BODB-Fe		DODB-Fe	
	Mass%	Atom%	Mass%	Atom%	Mass%	Atom%	Mass%	Atom%
Cl	0.06	0.10	—	—	—	—	—	—
C	—	—	3.48	13.24	0.20	0.56	31.40	56.99
N	—	—	0.34	1.12	0.04	0.05	3.02	4.68
O	—	—	3.39	9.69	27.91	57.11	12.66	17.25
S	—	—	0.06	0.10	0.36	0.37	1.46	1
Fe	99.94	99.90	92.73	75.85	71.49	41.91	51.46	20.08
**Total**	**100**	**100**	**100**	**100**	**100**	**100**	**100**	**100**

### 3.6. SRB biological resistivity

The SRB (sulfate-reducing bacteria) source was a water sample coming from an Egyptian gas field. We have previously discussed the water analysis, SRB population and monitoring procedures carried out using SRB (BART) vials (capacity = 15 ml).^41^ A small concentration (1 ppm mol^−1^) from each inhibitor was prepared in ultra-pure water and only 1 ml was added to the SRB test vial in addition to the water sample (15 ml) containing the SRB source. Another vial with only 15 ml of SRB water source without any added inhibitors was prepared as a blank. All vials were incubated at 35 °C inside an incubator. According to the test procedures, the maximum SRB test period is only 11 days but could be less according to realizing the first black sign had appeared on the test vials. After only 4 days, the test was completed for the blank with an aggressive population value of approximately 27 000 (cfu ml^−1^). For BODB, the test was completed after 7 days giving 325 (cfu ml^−1^) as the population value, with conversion of the severity of SRB to moderate instead of aggressive in the case of the blank sample. For both PhODB and DODB, the observed values were obtained after 8 days with a high effectiveness against SRB bacteria, giving 75 cfu ml^−1^ population (not aggressive), as listed in Table 10. The results clearly provide a valuable indication of a reduction in SRB reactivity. Furthermore, due to the biological activity of PhODB, BODB and DODB inhibitors, the corrosion resulting from the presence of SRB can be mitigated.

**Table 10:** Approximate SRB population for tested inhibitors

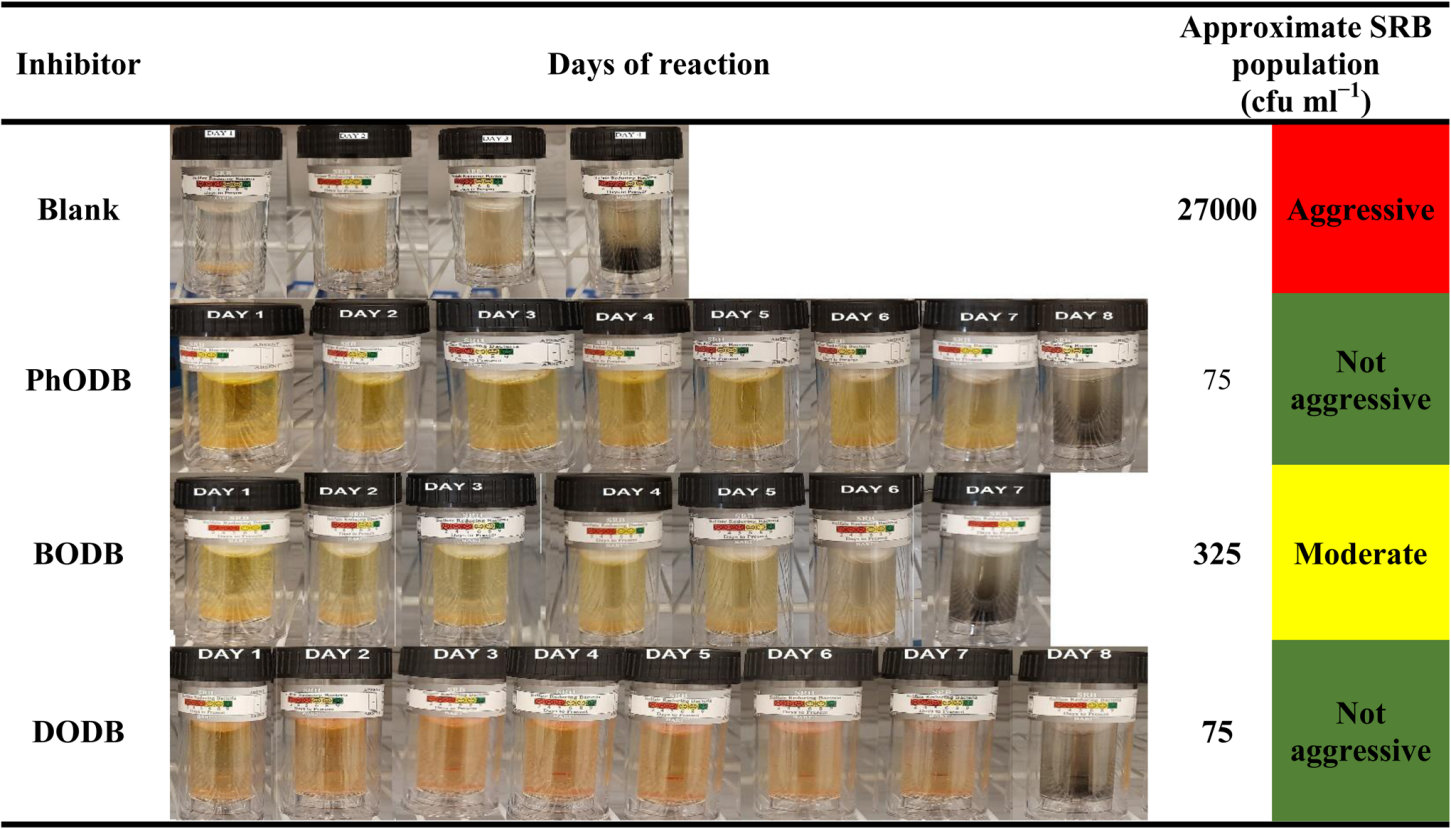

### 3.7. The correlation between quantum chemical calculations and the corrosion parameters

**Fig. 16:**
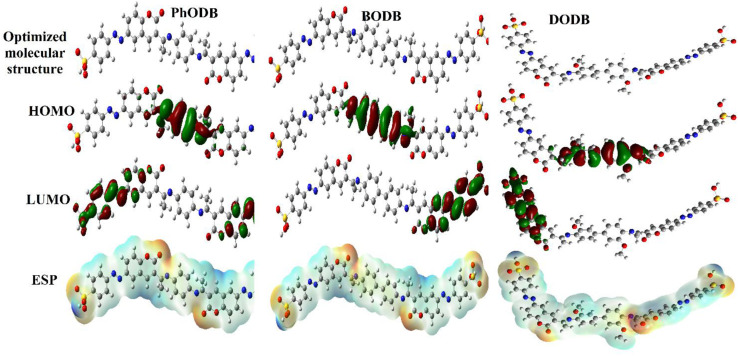
Optimized structures, HOMO, LUMO and ESP for synthesized coumarin derivatives (PhODB, BODB and DODB) using DFT/B3LYP/6-311+G.

**Fig. 17:**
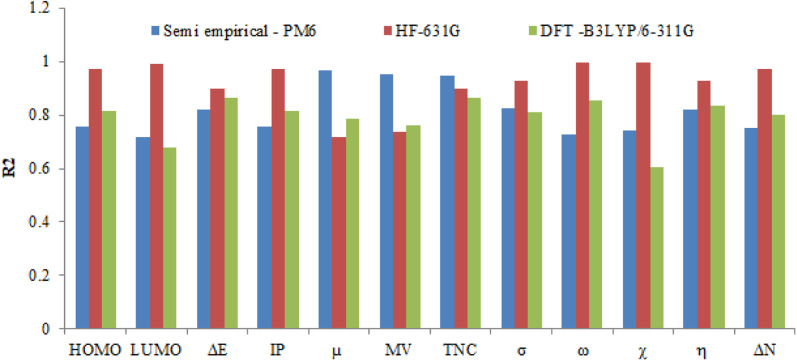
The regression value (*R*^2^) obtained from correlation between the calculated quantum chemical parameters and *η*_EFM_% for the synthesized coumarin derivatives (PhODB, BODB and DODB).

Using different basis sets (Semi-empirical PM6, HF-631G and DFT/B3LYP/6-311+G), quantum chemical calculations were carried out to investigate the reactivity, adsorption and interaction behavior between the inhibitors (PhODB, BODB and DODB) and MS.^81,82^ Many parameters were calculated using the basis sets (Semi-empirical PM6, HF-631G and DFT/B3LYP/6-311+G): highest occupied and lowest unoccupied molecular orbitals (*E*_HOMO_ and *E*_LUMO_, respectively), energy gap (Δ*E*), ionization potential (IP), electron affinity (EA), electronegativity (*χ*), electrophilicity (*ω*), transferred electrons (Δ*N*), softness (*σ*), hardness (*η*), dipole moment (*μ*), total energy *E* (RB3LYP), molecular volume (MV) and total negative charge (TNC), as listed in Table 11. Furthermore, the optimized molecular structures, HOMO, LUMO and ESP (electrostatic potential) resulting from (DFT/B3LYP/6-311+G) calculations for PhODB, BODB and DODB inhibitors are displayed in Fig. 16. An organic molecule's ability to donate electrons is generally demonstrated *via* its HOMO. In general, a molecule's ability to donate electrons is stronger with a higher *E*_HOMO_ value. Due to the lone pair of electrons as a result of heteroatoms and π electron as a result of the aryl ring there is greater capability for electron donation, and Fig. 16 shows that the HOMO is much more localized toward them.^83–87^ From the values listed in Table 11, the *E*_HOMO_ values are equal to −8.8862, −8.8451 and −8.6592 eV using Semi-empirical PM6, −8.1895, −7.8587 and −7.7710 eV using HF-631G, and −5.9715, −5.8948 and −5.6592 eV using DFT/B3LYP/6-311+G for PhODB, BODB and DODB, respectively. From the abovementioned *E*_HOMO_ results, DODB has the highest *E*_HOMO_ values and so it is has the greatest ability to donate electrons to the MS surface, leading to the formation a protective layer on its surface which is stronger than for BODB or PhODB. The energy gap (Δ*E*) is the difference between *E*_LUMO_ and *E*_HOMO_ values, and molecules with lower energy gaps deposit on metal surfaces more successfully because of the lower ionization energy that results from the lower energy gap, which makes it easier to remove the electron from the final orbital of the molecule.^88^ Δ*E* values equal 7.0829, 7.0608 and 6.9960 eV using semi-empirical PM6, 8.4483, 8.1188 and 8.0837 eV using HF-631G, and 2.4107, 2.3328 and 2.1211 eV using DFT/B3LYP/6-311+G for PhODB, BODB and DODB, respectively. Furthermore, the ionization potential (IP) values equal 8.8862, 8.8451 and 8.6592 eV using semi-empirical PM6, 8.1895, 7.8587 and 7.7710 eV using HF-631G, and 5.9715, 5.8948 and 5.6592 eV using DFT/B3LYP/6-311+G for PhODB, BODB and DODB, respectively. From the abovementioned Δ*E* and IP results, DODB has the lowest values, then BODB and PhODB have the highest values for the two parameters and, according to these values, DODB will have the highest reactivity to adsorb on the MS surface as a more effective corrosion inhibitor than BODB which will be more active than the PhODB inhibitor. Similarly, the compound with the lowest electronegativity (*χ*) values is the compound that most easily donates electrons to the MS surface and the same concept is also applicable to the total negative charge (TNC). According to the results in Table 11, the electronegativity (*χ*) values are equal to 5.3447, 5.3147 and 5.1612 eV using semi-empirical PM6, 3.9654, 3.7993 and 3.7292 eV using HF-631G, and 4.7662, 4.7284 and 4.5986 eV using DFT/B3LYP/6-311+G for PhODB, BODB and DODB, respectively. In addition, the total negative charge (TNC) results equal −14.5915, −15.3029 and −16.2161 eV using semi-empirical PM6, −14.9896, −15.8206 and −17.2966 eV using HF-631G, and −10.4903, −11.1590 and −12.6524 eV using DFT/B3LYP/6-311+G for PhODB, BODB and DODB, respectively. Therefore, DODB is considered to be the compound with higher protection ability than BODB or PhODB. Also, softness (*σ*) and hardness (*η*) are two chemical parameters which are related to each other. Chemical hardness prevents chemical molecules from deforming, and global hardness is negatively correlated with softness, so the highest softness compounds have the lowest hardness values.^89^ From the values in Table 11, the softness (*σ*) values are equal to 0.2824, 0.2823 and 2859 eV^−1^ using semi-empirical PM6, 0.2367, 0.2463 and 0.274 eV^−1^ using HF-631G, and 0.8296, 0.8573 and 0.9424 eV^−1^ using DFT/B3LYP/6-311+G for PhODB, BODB and DODB, respectively. In addition, the hardness (*η*) results are equal to 3.541, 3.530 and 3.498 eV using semi-empirical PM6, 4.224, 4.059 and 4.041 eV using HF-631G, and 1.205, 1.166 and 1.060 eV using DFT/B3LYP/6-311+G for PhODB, BODB and DODB, respectively. It is clear that the DODB compound has the highest softness values, and also the lowest hardness values and the highest ability to protect the MS surface, but PhODB has the lowest softness values, the highest hardness values and the lowest ability to protect the MS surface and finally BODB lies in between DODB and PhODB. Referring to ESP in Fig. 16, the electrophilic and nucleophilic reactivity can be predicted through the change in the color of the region: the blue color refers to nucleophilic reactivity and a positive region, but red and yellow colors refer to electrophilic reactivity and negative regions.^90^ Furthermore, the ability of the inhibitor to protect the metal surface increases with the increasing molecular volume (MV) of the inhibitor. From the values in Table 11, DODB has a higher MV than BODB or PhODB by using different calculation methods: semi-empirical PM6, HF-631G and DFT/B3LYP/6-311+G. Also, the number of electrons transferred (Δ*N*) provided good proof of the inhibitor's ability to donate electrons to the metal surface, and from the obtained results DODB has the highest ability for electron donation, but PhODB has the lowest ability. The unshared electrons can act as a Lewis base and be easily donated to the metal ion (acting as a Lewis acid) *via* the vacant d orbital. By sharing the electrons from the inhibitor to the vacant d orbitals on the metal ion, a coordination bond is easily formed, resulting in complex formation between the inhibitor and the metal surface. The result is protection of the metal from attack by the corrosive electrolyte.^91–93^ The regression values (*R*^2^) for the calculated quantum chemical parameters and the *η*_EFM_% are plotted in Fig. 17 and listed in Table 12. The values resulting from the theoretical quantum chemical calculation are in a good agreement with the values from the experimental results and suggest the inhibition order is DODB > BODB > PhODB.

**Table 11:** The calculated quantum chemical parameters using 3 different optimization basis sets: semi-empirical PM6, HF-631G and DFT/B3LYP/6-311G

OPT	Molecule	*E*_HOMO_ (eV)	*E*_LUMO_ (eV)	Δ*E* (eV)	IP (eV)	*μ* (D)	MV (cm^3^ mol^−1^)	TNC (e)	*σ* (eV^−1^)	*ω* (eV)	*χ* (eV)	*η* (eV)	Δ*N* (e)	*η*_PDP_%
Semi-empirical PM6	PhODB	−8.8862	−1.8033	7.0829	8.8862	0.9283	631.388	−14.5915	0.2824	4.0331	5.3447	3.541	0.2337	95.29
	BODB	−8.8451	−1.7843	7.0608	8.8451	4.5997	814.211	−15.3029	0.2833	4.0003	5.3147	3.530	0.2387	96.50
	DODB	−8.6592	−1.6632	6.9960	8.6592	5.4661	846.2280	−16.2161	0.2859	3.8076	5.1612	3.498	0.2628	97.60
HF-631G	PhODB	−8.1895	0.2588	8.4483	8.1895	2.8506	545.8450	−14.9896	0.2367	1.8612	3.9654	4.224	0.3592	95.29
	BODB	−7.8587	0.2601	8.1188	7.8587	2.7910	559.3780	−15.8206	0.2463	1.7779	3.7993	4.059	0.3942	96.50
	DODB	−7.7710	0.3127	8.0837	7.7710	2.4156	631.3660	−17.2966	0.2474	1.7204	3.7292	4.041	0.4046	97.60
DFT/B3LYP/6-311G	PhODB	−5.9715	−3.5609	2.4107	5.9715	0.0024	537.5310	−10.4903	0.8296	9.4235	4.7662	1.205	0.9266	95.29
	BODB	−5.8948	−3.5620	2.3328	5.8948	0.7567	571.0750	−11.1590	0.8573	9.5839	4.7284	1.166	0.9738	96.50
	DODB	−5.6592	−3.5380	2.1211	5.6592	3.5620	719.8810	−12.6524	0.9429	9.9697	4.5986	1.060	1.1321	97.60

**Table 12:** The calculated *R*^2^ values using different optimization basis sets: semi-empirical PM6, HF-631G and DFT/B3LYP/6-311G

Optimized basis sets	*E*_HOMO_ (eV)	*E*_LUMO_ (eV)	Δ*E* (eV)	IP (eV)	*μ* (D)	MV (cm^3^ mol^−1^)	TNC (e)	*σ* (eV^−1^)	*ω* (eV)	*χ* (eV)	*η* (eV)	Δ*N* (e)
Semi-empirical PM6	0.7583	0.7188	0.8204	0.7583	0.9674	0.9507	0.9468	0.8228	0.727	0.7429	0.8217	0.7502
HF-631G	0.9738	0.9904	0.9003	0.9738	0.7196	0.7383	0.9003	0.9294	0.9969	0.9952	0.9273	0.9728
DFT/B3LYP/6-311G	0.8133	0.6803	0.8639	0.8133	0.7849	0.7608	0.8639	0.8123	0.8519	0.6052	0.8324	0.8000

## 4. Conclusions

Three novel coumarin derivatives were synthesized and characterized by different analyses. The inhibition efficiency increases when the inhibitor concentration and temperature of the environment are raised, which indicates that the adsorption is mainly chemical. Adsorption of these derivatives onto the MS surface in 1 M HCl solution obeys the Langmuir adsorption model. Potentiodynamic polarization studies reveal that these derivatives are mixed-type inhibitors. Electrochemical impedance measurements indicate the formation of a protective film on the MS surface in HCl solution. FTIR spectroscopic data suggest that the protective film consists of an Fe–additive molecule complex. SEM and XRD analyses clearly indicate the presence of a protective surface layer on the MS surface. The results showed that corrosion related to SRB can be controlled by these novel coumarin derivatives. Theoretical calculation show an amazing match with the experimental results. The suggested inhibition order according to the resulting values from theoretical and experimental techniques is as follows: DODB > BODB > PhODB.

## Author contributions

Hani M. Elaryian: methodology and taking experiment part of inhibitors synthesized and tested, writing – original draft preparation. Mahmoud A. Bedair: supervision, software, resources, conceptualization, experimental, validation, formal analysis, FMO computations, review and editing article. Ahmed H. Bedair: supervision, resources. Rabab M. Aboushahba: supervision. Abd El-Aziz S. Fouda: supervision, conceptualization, investigation, software, validation, review and editing article.

## Conflicts of interest

The authors declare that they have no known competing financial interests or personal relationships that could have appeared to influence the work reported in this paper.

## Supplementary Material
